# A prior-knowledge-guided feature selection method and its application to biomarker identification of schizophrenia

**DOI:** 10.1162/NETN.a.37

**Published:** 2025-11-20

**Authors:** Ying Xing, Godfrey D. Pearlson, Peter Kochunov, Vince D. Calhoun, Yuhui Du

**Affiliations:** School of Computer and Information Technology, Shanxi University, Taiyuan, China; Departments of Psychiatry and Neurobiology, Yale University, New Haven, CT, USA; Faillace Department of Psychiatry and Behavioral Sciences at McGovern Medical School, The University of Texas Health Science Center, Houston, TX, USA; Tri-Institutional Center for Translational Research in Neuroimaging and Data Science, Georgia State University, Georgia Institute of Technology, Emory University, Atlanta, GA, USA

**Keywords:** Dimensionality reduction, Classification, Mental disorders, Functional network connectivity, Biomarker

## Abstract

Despite considerable efforts to uncover the neural basis of psychiatric disorders using neuroimaging, few methods utilize intrinsic brain-derived knowledge, leading to limited specificity and discriminability in biomarker identification. To leverage the inherent characteristics within the brain, we propose a prior-knowledge-guided feature selection method to flexibly unveil discriminative and target-oriented biomarkers of psychiatric disorders. Specifically, we construct a constrained sparse regularization allowing for the flexible integration of diverse prior knowledge to identify sparse neuroimaging features linked to specific psychopathology. Additionally, we simultaneously integrate graph-based regularization and redundancy-removal regularization to further ensure the discriminability and independence among the selected features. Different priors hold varying significance in identifying specific biomarkers. Four functional magnetic resonance imaging (fMRI) datasets from 708 healthy controls and 537 schizophrenia patients are used to evaluate our method integrated with various prior knowledge, revealing specific schizophrenia-related brain abnormalities. Compared with nine advanced feature selection methods, our method improves mean classification accuracy by 3.89% to 11.24%, particularly revealing reduced interactions within the visual domain and between subcortical and visual domains in schizophrenia patients. The proposed method offers flexible and precise biomarker identification tailored to specific targets, advancing the understanding and diagnosis of psychiatric conditions.

## Author Summary

We propose a prior-knowledge-guided feature selection (PriFS) method to identify discriminative and target-oriented biomarkers for psychiatric disorders by flexibly integrating diverse brain-derived knowledge. PriFS combines constrained sparse regularization with graph-based and redundancy-removal regularization terms to enhance feature specificity, discriminability, and independence. Evaluated on fMRI data from 708 healthy controls and 537 schizophrenia patients, PriFS outperforms nine comparison methods, improving classification accuracy by 3.89%–11.24%. It reveals schizophrenia-specific abnormalities, including reduced interactions within the visual domain and between the subcortical and visual domains. By leveraging varying prior knowledge, PriFS uncovers important and specific biomarkers, advancing the understanding and diagnosis of psychiatric conditions.

## INTRODUCTION

As the most complex organ in the human body, the brain mediates cognitive functions through precisely coordinated neural network interactions. In psychiatric disorders, aberrations in these neurophysiological processes lead to dysregulation of neural networks, imposing substantial health care and economic burdens (Scangos, State, Miller, Baker, & Williams, 2023). This reality necessitates the development of objective biomarkers—quantifiable biological indicators that reliably reflect neuropathological mechanisms or therapeutic responses (Blomsma et al., 2022). Resting-state functional magnetic resonance imaging (rs-fMRI) has demonstrated particular promise in discovering potential disease-specific biomarkers, which may enable presymptomatic detection in major psychiatric disorders (Cwiek et al., 2022; Xing, van Erp, et al., 2024).

The complex and high-dimensional nature of rs-fMRI data presents a significant challenge in directly uncovering the underlying mechanisms of brain function abnormalities in patients with psychiatric disorders. Currently, supervised feature selection methods are pivotal in discovering psychiatric biomarkers, as they not only effectively reduce the dimensionality of neuroimaging data but also reveal the pathophysiological mechanisms of psychiatric disorders (Xing, Pearlson, Kochunov, Calhoun, & Du, 2024). For instance, Karavallil Achuthan, Coburn, Beckerson, and Kana (2023) used statistical tests to reveal abnormal brain functions in children with autism. Utilizing data from both structural and functional modalities, Chen et al. (2024) uncovered distinct neurobiological markers for schizophrenia (SZ) and bipolar disorder using the recursive feature elimination (RFE) method, highlighting the superior accuracy of rs-fMRI measures in classification tasks. Yamashita et al. (2020) used the least absolute shrinkage and selection operator (Lasso) method to discover decreased functional connectivity between brain regions in patients with major depressive disorder compared with healthy controls (HCs). Additionally, we previously proposed a local-structure-preservation and redundancy-removal-based feature selection (LRFS) method (Xing, Pearlson, et al., 2024), identifying more discriminative and clinically relevant functional biomarkers for SZ. However, these general feature selection methods may overlook the intrinsic meaning within intricate neuroimaging data, leading to the selected features with inadequate interpretability.

The incorporation of domain knowledge serves as an effective strategy to prioritize features that are not only discriminative but also interpretable and aligned with real-world constraints (Teufel & Fletcher, 2020; Yousef, Kumar, & Bakir-Gungor, 2020). In material science, Liu, Zou, Ma, Avdeev, and Shi (2022) incorporated physicochemical constraints to prevent the selection of unreasonable material features, ensuring domain-consistent predictions. For photovoltaic forecasting, Luo, Zhang, and Zhu (2021) embedded physical constraints into model training, penalizing unrealistic outputs (e.g., negative power generation) to improve reliability. In antibody engineering, Clark et al. (2023) leveraged protein interaction knowledge to design biologically relevant features for mutation prediction. Similarly, in gene expression analysis, Qi and Tang (2007) combined gene ontology annotations with statistical metrics to select discriminative yet biologically plausible biomarkers. In neuroimaging and biomarker identification, domain knowledge plays a crucial role in refining feature selection. For example, whole-brain functional networks can be subdivided into positive and negative connectivity with respect to synergistic and inhibitory relationships between brain regions, focusing on revealing specific patterns linked to brain traits, diseases, aging, and gender (Mijalkov et al., 2023; Tse et al., 2024). Other studies use expert knowledge to preselect regions or features of interest, enhancing subsequent feature selection and analysis (Pan et al., 2022). Introducing relevant prior knowledge facilitates the precise identification of biomarkers tailored to specific targets, paving the way for unveiling new discoveries and enhancing the logical coherence of the findings. However, conventional fMRI feature selection methods fail to effectively incorporate prior knowledge (e.g., known brain region functions or established disease-related pathways) into the feature selection process. This limitation not only hinders the identification of discriminative neuroimaging features aligned with specific objectives but also overlooks biologically meaningful features that could be highlighted through guided selection.

To more effectively integrate prior knowledge with feature selection within a unified framework, we propose a novel prior-knowledge-guided feature selection (PriFS) method that aims to flexibly identify target-oriented, discriminative, and low-redundancy biomarkers. Specifically, we incorporate domain-specific prior knowledge into the *l*_2,1_-norm sparse regularization through a constraint matrix that differentially penalizes features according to their relevance to the target condition. Domain-consistent features receive smaller penalties to preserve domain relevance, whereas domain-irrelevant features receive larger penalties, thereby promoting sparsity and interpretability. This approach enables the model to flexibly prioritize biologically plausible and interpretable biomarkers. Additionally, the graph-based regularization term preserves intrinsic data similarity structures, and the redundancy-removal term reduces the influence of correlated and redundant features, further improving discriminability and reducing redundancy. SZ is a complex psychiatric disorder characterized by widespread dysconnectivity across multiple functional circuits. The incorporation of prior knowledge into feature selection methods allows for more targeted identification of SZ-related abnormalities. To validate our method guided by varied prior knowledge, we employ functional network connectivity (FNC) of multisite rs-fMRI data including SZ patients and age-matched HCs to identify specific SZ-related abnormalities.

Our contributions can be summarized as follows:

(1)  PriFS method employs a straightforward yet effective strategy to construct a constrained *l*_2,1_ sparse regularization term, which integrates various prior knowledge derived from brain characteristics. Consequently, it effectively and flexibly uncovers sparse and target-oriented features. Additionally, by incorporating the graph-based and redundancy-removal regularization terms, PriFS method further ensures the discriminability of the selected features and independence among the features.
(2)  Comprehensive experiments conducted on multisite rs-fMRI datasets support the superior performance of our method informed by diverse prior knowledge. Our method attains average improvements in classification accuracy of 3.89% to 11.24% over nine state-of-the-art methods, highlighting the advantage of our method in distinguishing SZ patients from HCs.
(3)  Our method using diverse prior knowledge yields distinct features with improved discriminability, enhancing the multifaceted understanding of SZ. It demonstrates favorable scalability, allowing for the flexible integration of additional prior knowledge, such as various brain connectivity data, brain topological structures, and behavioral information.

## RELATED WORK

Feature selection methods can be broadly categorized into filter, wrapper, and embedded methods (Du, Niu, Xing, Li, & Calhoun, 2025), as detailed in Table 1. Filter methods evaluate feature importance based on intrinsic data characteristics, without relying on any specific model. For example, the ReliefF method (Kononenko, Šimec, & Robnik-Šikonja, 1997) assigns importance weights to features by assessing their ability to distinguish between neighboring samples. The analysis of variance (ANOVA) method (Ferdinandov et al., 2023) selects features with significant intergroup differences by calculating the F-statistic for each feature. Information theory-based methods such as minimum redundancy maximum relevance (mRMR) (Peng, Long, & Ding, 2005) select features that simultaneously minimize inter-feature redundancy and maximize feature-class relevance. Collectively, filter methods are widely used in biomarker identification due to their speed and computational simplicity, but their lack of interaction with classifiers may compromise the performance in complex tasks.

**Table 1.** Filter, wrapper, and embedded methods for feature selection

**Category**	**Characteristic**	**Representative algorithm**
Filter methods	Evaluate feature importance using data-derived metrics without model involvement.	ReliefF: evaluates feature importance by iteratively weighting each feature’s capacity to distinguish between nearest neighbor instances from the same and opposite classes. ANOVA: ranks features by computing F-statistics to identify variables with statistically significant differences across groups. mRMR: optimizes feature subsets by maximizing relevance to the target variable while minimizing inter-feature redundancy through mutual information metrics.
Wrapper methods	Evaluate feature importance by assessing model performance across different feature subsets.	RFE: iteratively eliminates the least important features based on model-derived importance rankings (i.e., classification accuracy). SIFE: combines evolutionary optimization with set operations and fuzzy granulation strategies to evolve integer-coded feature subsets that balance discriminability and diversity. GA: mimics natural evolution by applying selection, crossover, and mutation operations to population-encoded feature subsets, optimizing classification performance.
Embedded methods	Evaluate feature importance during model training based on their contribution to model performance.	Lasso: enforces feature sparsity through *l*_1_-norm regularization, performing simultaneous feature selection and linear regression by minimizing reconstruction error. Elastic net: combines *l*_1_ and *l*_2_ regularization to balance feature sparsity and group effect, addressing multicollinearity while selecting discriminative features. RFS: employs *l*_2,1_-norm to measure transformation error, achieving noise-resistant feature selection by jointly suppressing irrelevant features and outlier sensitivity. FS20: directly selects top-*k* features through explicit *l*_2,0_-norm constraints, bypassing relaxation approximations used in conventional sparse learning. *l*_2,1_-RLDA: reformulates LDA with *l*_2,1_-norm regularization to suppress noisy features while preserving class-discriminative structures in the projected subspace. S^2^DFS: integrates trace-ratio criterion with *l*_2,0_-norm structural sparsity to select features that optimize both subspace discrimination and subset compactness. ALLDA: adaptively constructs sample-specific neighborhoods to learn locality-preserving transformations, enhancing discriminability in local manifolds. LRFS: jointly imposes graph Laplacian regularization and feature redundancy constraints to select maximally discriminative and mutually independent features.

Wrapper methods determine feature importance through the predictive performance of a specific model. A classic example is the RFE method (Tian et al., 2022), which progressively eliminates less contributive features through iterative model retraining, ultimately identifying the most discriminative features for the model. The set-based integer-coded fuzzy-granular evolutionary (SIFE) method (Saadatmand & Akbarzadeh-T, 2023) incorporates set operations and fuzzy granulation strategies into evolutionary algorithms, identifying the optimal feature set by measuring the similarity and dissimilarity between diverse feature subsets. Inspired by biological evolution principles, genetic algorithms (GAs) (Oh, Lee, & Moon, 2004) efficiently navigate search spaces using natural selection mechanisms to evolve feature subsets that maximize model accuracy. While wrapper methods typically deliver superior predictive accuracy compared with filter methods, this advantage comes at the cost of substantial computational overhead due to repeated model training.

Embedded methods integrate feature selection into model construction by optimizing an objective function that both reduces the empirical risk of classification and promotes feature sparsity (Carrasco, Ivorra, López, & Ramos, 2024). For example, the Lasso method (Tibshirani, 1996) is a classical feature selection method that aims to minimize the mean squared error before and after data transformation while enforcing feature sparsity via *l*_1_-norm. The elastic net method (Zou & Hastie, 2005) combines *l*_1_ and *l*_2_ regularization to achieve sparse feature selection while maintaining group stability for correlated features. Considering the sensitivity of the *l*_1_-norm to noise, the robust feature selection (RFS) method (Nie, Huang, Cai, & Ding, 2010) uses the *l*_2,1_-norm to measure transformation error and enforce sparsity, thus selecting a sparse and robust feature subset even in noisy data. The feature selection via *l*_2,0_-norm constraint (FS20) method (X. Cai, Nie, & Huang, 2013) directly selects the top *k* relevant features by imposing an *l*_2,0_-norm constraint. The *l*_2,1_-norm robust linear discriminant analysis feature selection (*l*_2,1_-RLDA) method (Nie, Wang, Wang, Wang, & Li, 2021) constructs a robust LDA feature selector with *l*_2,1_-norm, mitigating the impact of noisy features on traditional LDA methods. The subspace discriminant sparse feature selection (S^2^DFS) method (Wang, Nie, Tian, Wang, & Li, 2020) combines the trace ratio criterion with structural sparsity in subspace constraints using the *l*_2,0_-norm to select a sparse feature subset. The adaptive local linear discriminant analysis (ALLDA) method (Nie, Wang, Wang, Wang, & Li, 2020) adaptively assigns *k*-nearest neighbors to each sample and learns the similarity matrix and transformation matrix that maintains the local structure of the data. The LRFS method (Xing, Pearlson, et al., 2024) combines graph-based and redundancy-removal regularizations to discover more discriminative and independent features. Although these methods balance both effectiveness and efficiency, they do not fully leverage the inherent prior knowledge of brain data and leave room for improvement in terms of interpretability.

## METHODS

We propose a PriFS method, aiming at flexibly pinpointing discriminative, target-oriented, and specific neuroimaging biomarkers for psychiatric disorders. This method capitalizes on the inherent characteristics of the brain and effectively preserves the local structural information of the data while promoting independence among selected features, thereby improving the method’s interpretability and discriminability. The realization of these goals is facilitated by the integration of three specialized regularization terms, including constrained *l*_2,1_ sparse regularization, graph-based regularization, and redundancy-removal regularization. Each term plays a vital role in enhancing the overall efficacy of the model. Additionally, we theoretically prove that the proposed objective function possesses an optimal solution in this study.

### Constrained *l*_2,1_ Sparse Regularization Term to Capture Specific Patterns in Brain Property

Regularization model-based feature selection methods aim to identify a sparse subset of discriminative features. These methods typically take the form of an optimization problem, which includes a fitting error term and a sparse regularization (Tibshirani, 1996). Here, we propose a novel constrained *l*_2,1_ sparse regularization to flexibly incorporate established prior knowledge into feature selection method. Such integration is particularly conducive to the selection of specific, robust, and target-oriented features with enhanced discriminability, which is crucial for the personalized diagnosis of psychiatric disorders. It is important to note that we employ *l*_2,1_-norm as it has been proven to significantly enhance both the robustness of the model and the sparsity of the selected features (Mo & Lai, 2019).

For a given data matrix ***X*** = [***x***_1_, ***x***_2_, …, ***x****_n_*]*^T^* ∈ ℝ^*n*×*d*^, ***x****_i_* ∈ ℝ*^d^* represents the feature vector for the *i*th sample, where *n* and *d* denote the number of samples and the number of features, respectively. Correspondingly, for a given label matrix ***Y*** = [***y***_1_, ***y***_2_, …, ***y****_n_*]*^T^* ∈ ℝ^*n*×*c*^, ***y****_i_* = [*y*_*i*1_, *y*_*i*2_, …, *y*_*ic*_] ∈ {−1, 1}^1×*c*^ (*c* is the number of groups) represents the label vector for sample ***x****_i_*, with *y*_*ij*_ = 1 if ***x****_i_* belongs to the *j*th group (*j* ∈ {1, …, *c*}) and *y*_*ij*_ = −1 otherwise. The constrained *l*_2,1_ sparse regularization-based feature selection method is formulated to solve the following optimization problem:

$$\text{arg}\min_{W}{\left\|Y-XW\right\|}_{F}^{2}+\lambda {\left\|B⊙W\right\|}_{2,1}.$$ (1)

The first term represents the fitting error, where ***W*** ∈ ℝ^*d*×*c*^ is the data transformation matrix indicating the importance of features. The second term is constrained *l*_2,1_ sparse regularization to promote the selection of sparse, robust, and specific features. Matrix ***B*** ∈ ℝ^*d*×*c*^ serves as a constraint matrix informed by prior knowledge to calibrate the data transformation matrix ***W***. Specifically, ***B*** is constructed to mitigate penalties on target-oriented features, consequently amplifying their associated feature importance vectors ${\hat{w}}_{i}$. Conversely, ***B*** imposes greater penalties on features that are not target-oriented features, thereby diminishing the corresponding vectors ${\hat{w}}_{i}$. ${\hat{w}}_{i}$ (*i* ∈ {1, …, *d*}) is the *i*th row of matrix ***W*** and conveys the importance of the *i*th feature. Symbol ⊙ is Hadamard product between matrices (i.e., element-wise multiplication between matrices). The regularization parameter *λ* balances the tradeoff between the fitting error and sparsity.

Matrix ***B*** can be sourced either from the data itself or from various modalities, and can be flexibly integrated into the objective function. For example, one might construct the constraint matrix ***B*** using functional connectivity patterns ascertained from rs-fMRI data, which reveal the distinct interactions between functional networks. Alternatively, the construction of ***B*** can be informed by established correlations between neuroimaging features and behavioral data. In short, the constrained *l*_2,1_ sparse regularization flexibly leverages existing prior knowledge to enhance the interpretability of selected features, focusing on the selection of sparse, significant, and target-oriented features.

### Graph-Based Regularization to Preserve Local Structure of Data

We anticipate that samples which are similar in the original feature space will retain their similarity in the transformed space, as suggested by previous work (Li, Chen, Li, Wan, & Sang, 2022). To achieve this, we focus on minimizing the following expression:

$$\frac{1}{2}\sum_{i=1}^{n}\sum_{j=1}^{n}{\left\|x_{i}W-x_{j}W\right\|}_{2}^{2}S_{ij}=Tr\left(W^{T}X^{T}LXW\right),$$ (2)

where *S*_*ij*_ in the sample similarity matrix ***S*** quantifies the similarity relationship between neighboring samples ***x****_i_* and ***x****_j_*. If ***x****_i_* is one of the *k*-nearest neighbors of ***x****_j_* or vice versa, *S*_*ij*_ = exp (−‖***x****_i_* − ***x****_j_*‖^2^/2*σ*^2^); otherwise, *S*_*ij*_ = 0. The neighborhood parameter k is automatically set to the square root of half the sample size, ensuring adaptive scaling with the dataset. This setting reduces manual intervention while balancing local structure preservation and computational efficiency. For simplicity, we set the heat kernel parameter *σ*^2^ = 0.5. ***L*** = ***D*** − ***S*** is the Laplacian matrix and ***D*** is the diagonal matrix with $D_{ii}=\sum_{j=1}^{n}S_{ij}$. By minimizing the above expression, similar pairs of samples ***x****_i_* and ***x****_j_* (with a large value of *S*_*ij*_) in the original high-dimensional space remain similar in the transformed space with a small value of ${\left\|x_{i}W-x_{j}W\right\|}_{2}^{2}$. It should be noted that, due to the semipositive definiteness of ***L***, minimizing the graph-based regularization constitutes a convex optimization problem.

### Redundancy-Removal Regularization to Eliminate the Negative Impact of Redundant Features

Feature redundancy stemming from similar features adversely affects the efficacy of feature selection methods. In neuroimaging data, such redundancies can obscure the precise detection of biomarkers, necessitating the incorporation of a regularization term within the objective function to promote independence among selected features. While the constrained *l*_2,1_ sparse regularization promotes feature-level sparsity, it does not explicitly account for inter-feature redundancy. Therefore, to further ensure that the selected features are independent and low redundant, we introduce a similarity-based regularization term that penalizes the simultaneous selection of important but analogous features. This objective is achieved by minimizing the following expression:

$$\sum_{i=1}^{d}\sum_{j=1}^{d}\left(w_{i}{w_{j}}^{T}\right)Q_{ij}=Tr\left(W^{T}QW\right),$$ (3)

where *Q*_*ij*_ represents the similarity of the *i*th feature (i.e., ***f****_i_*) with *j*th feature (i.e., ***f****_j_*), and is defined as

$$Q_{ij}={\left(\frac{{f_{i}}^{T}˙f_{j}}{\left\|f_{i}\|˙\|f_{j}\right\|}\right)}^{2}={\left(\frac{\sum_{k=1}^{n}f_{ik}˙f_{jk}}{\sqrt{\sum_{k=1}^{n}{f_{ik}}^{2}}˙\sqrt{\sum_{k=1}^{n}{f_{jk}}^{2}}}\right)}^{2}$$ (4)

Minimizing Equation 3 enforces the selection of mutually independent features. It’s important to highlight that the introduction of the redundancy-removal regularization term in the objective function remains tractable during the optimization process, thanks to the semi-positive definiteness of the feature similarity matrix ***Q***.

### Final Objective Function and Optimization

In summary, the graph-based regularization term (i.e., Equation 2) and the redundancy-removal regularization term (i.e., Equation 3) are integrated in Equation 1, which incorporates the constrained *l*_2,1_ sparse regularization term, to construct the final objective function. The collective formulation preserves the local structural information of data, ensures the independence among features, and identifies sparse and discriminative features with specific targets. Therefore, the final objective function is as follows:

$$\text{arg}\min_{W}{\left\|Y-XW\right\|}_{F}^{2}+\lambda_{1}Tr\left(W^{T}X^{T}LXW\right)+\lambda_{2}Tr\left(W^{T}QW\right)+\lambda_{3}{\left\|B⊙W\right\|}_{2,1},$$ (5)

where *λ*_1_, *λ*_2_, and *λ*_3_ are regularization parameters to balance the tradeoff of the corresponding regularization terms. A nonnegative linear combination of convex functions preserves convexity (Boyd & Vandenberghe, 2004; Niculescu & Persson, 2006). The objective function in Equation 5, denoted as *J*(***W***), is jointly convex with respect to ***W***, since it represents a nonnegative linear combination (with *λ*_1_, *λ*_2_, *λ*_3_ ≥ 0) of four convex terms, thereby ensuring the global optimality of the solution.

Here, we present an efficient iterative algorithm to solve the optimization problem associated with the objective function *J*(***W***). Since it is difficult to directly differentiate with respect to ***W***, we differentiate *J*(***W***) with respect to *W*_*ij*_ that is the element in the *i*th row and *j*th column of matrix ***W***:

$$\frac{\partial J\left(W\right)}{\partial W_{ij}}={X_{ij}}^{T}X_{ij}W_{ij}-{X_{ij}}^{T}Y_{ij}+\lambda_{1}{X_{ij}}^{T}L_{ij}X_{ij}W_{ij}+\lambda_{2}Q_{ij}W_{ij}+\frac{\lambda_{3}}{2}\frac{{\left(B⊙B⊙W\right)}_{ij}}{{\left(\sum_{j=1}^{c}{\left(B_{ij}W_{ij}\right)}^{2}\right)}^{\frac{1}{2}}}.$$ (6)

Let ***U*** represent a diagonal matrix, which serves as an intermediate variable calculated from ***W***:

$$U=\text{diag}\left({\left(\sum_{j=1}^{c}{\left(B_{1j}W_{1j}\right)}^{2}\right)}^{-\frac{1}{2}},\ldots ,{\left(\sum_{j=1}^{c}{\left(B_{dj}W_{dj}\right)}^{2}\right)}^{-\frac{1}{2}}\right).$$ (7)

Let $\frac{\partial J\left(W\right)}{\partial W}=0$, Equation 6 can be written as:

$$\frac{\partial J\left(W\right)}{\partial W}=\left(2X^{T}XW-2X^{T}Y\right)+2\lambda_{1}X^{T}LXW+2\lambda_{2}QW+\lambda_{3}\left(U\left(B⊙B⊙W\right)\right)=0.$$ (8)

According to Equation 8, it is still difficult to solve for ***W*** as a whole. Therefore, we seek the optimal W^*^ by solving for each column in ***W***, resulting in ***W***^∗^ = [***W***_1_^*^, ***W***_2_^*^, …, ***W***_c_^*^]. For the *j*th column ***W****_j_* in ***W***:

$$X^{T}XW_{j}+\lambda_{1}X^{T}LXW_{j}+\lambda_{2}QW_{j}+\frac{\lambda_{3}}{2}U\left(B_{j}⊙B_{j}⊙W_{j}\right)=X^{T}Y_{j}.$$ (9)

Let diagonal matrix

$$M=\text{diag}\left\{\left(\frac{\lambda_{3}}{2}U_{11}\right){B_{1j}}^{2},\ldots ,\left(\frac{\lambda_{3}}{2}U_{dd}\right){B_{dj}}^{2}\right\}.$$ (10)

Then,

$$W_{j}={\left(X^{T}X+\lambda_{1}X^{T}LX+\lambda_{2}Q+M\right)}^{-1}\left(X^{T}Y_{j}\right).$$ (11)

The iterative procedure of PriFS method is summarized in Algorithm 1. Initially, the constraint matrix ***B*** is computed according to specific prior knowledge. Then, we update the diagonal matrix ***U***^(*t*)^ according to ***W***^(*t*)^ as described in Equation 7. During one iteration, the update of ***W***^(*t*+1)^ involves independently updating each column ***W****_j_*^(*t*+1)^, by computing the diagonal matrix ***M***^(*t*)^ as specified in Equation 10. Consequently, ***W***^(*t*+1)^ = [***W***_1_^(*t*+1)^, …, ***W****_c_*^(*t*+1)^] is updated. The entire iteration process halts upon achieving convergence, or reaching the predefined maximum number of iterations. It is important to highlight that the optimization problem defined by Equation 5 is strictly convex, which ensures the existence of a unique global optimal solution for the matrix ***W***. Detailed proofs for the convergence are given in the Supporting Information.


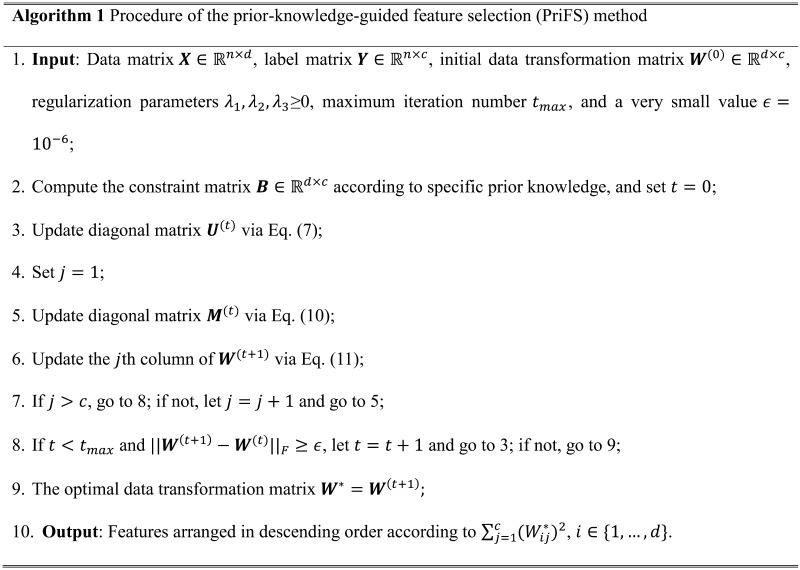


## EXPERIMENTS AND RESULTS

To validate the effectiveness of the proposed PriFS method in identifying discriminative biomarkers for psychiatric disorders, we compare it with nine state-of-the-art feature selection methods using rs-fMRI data of 537 SZ patients and 708 age-matched HCs. We employ three widely used classifiers, including support vector machine (SVM), 5-nearest neighbor (5NN), and LDA, to evaluate the discriminability of the features selected by each method, as they have demonstrated strong and efficient performance in neuroimaging-based classification tasks (Borchert et al., 2023; Du, Fu, & Calhoun, 2018). Additionally, the influence of different parameter settings on the performance of our method is examined empirically. We also perform ablation experiments to assess the contribution of each term in the objective function (i.e., Equation 5) in distinguishing between groups. Furthermore, we provide a detailed analysis of the important features selected by the proposed method as potential biomarkers, indicating abnormal brain functional connectivity associated with SZ.

### Data and Preprocessing

In this study, rs-fMRI data of 1,245 subjects are selected from four multisite datasets to evaluate the performance of the proposed method and explore potential biomarkers for SZ. Specifically, we utilize 68 SZ patients and 89 age-matched HCs from the Centers of Biomedical Research Excellence (COBRE), 150 SZ patients and 238 age-matched HCs from the Maryland Psychiatric Research Center (MPRC), 137 SZ patients and 144 age-matched HCs from the Function Biomedical Informatics Research Network (FBIRN), and 182 SZ patients and 237 age-matched HCs from the Bipolar-Schizophrenia Network on Intermediate Phenotypes phase I (B-SNIP). All human data used in this study are obtained from previously published studies that had been approved by their respective institutional review boards or relevant research ethics committees. Written consent from all subjects and ethics approval is also obtained. A detailed description of the demographic information is outlined in Table 2. In these four datasets, there are no significant intergroup differences (*p* ≤ 0.01 with Bonferroni correction) between the HC group and the SZ group in terms of age and head motion. Furthermore, on the COBRE and FBIRN datasets, the gender between the HC and SZ groups also does not show significant intergroup differences. We further eliminate their influence in the subsequent analysis.

**Table 2.** Demographic information of the participants

		**Subject number: male/female**	**Age: mean (*SD*)**	**Transitions: mean (*SD*)**	**Rotations: mean (*SD*)**
**COBRE**	**HC**	64/25	38.09 (11.66)	0.22 (0.15)	0.19 (0.12)
	**SZ**	57/11	37.74 (14.47)	0.20 (0.12)	0.18 (0.12)
	***p* value**	0.0785	0.8652	0.3624	0.5258
**MPRC**	**HC**	94/144	40.24 (15.17)	0.09 (0.06)	0.07 (0.06)
	**SZ**	98/52	38.7 (14.05)	0.10 (0.10)	0.08 (0.1)
	***p* value**	7.16e−07	0.3186	0.0617	0.2419
**FBIRN**	**HC**	104/40	37.15 (11)	0.19 (0.15)	0.21 (0.16)
	**SZ**	103/34	39.02 (11.35)	0.18 (0.13)	0.20 (0.16)
	***p* value**	0.5733	0.1605	0.4239	0.4223
**B-SNIP**	**HC**	137/100	38.13 (12.59)	0.13 (0.10)	0.13 (0.12)
	**SZ**	56/126	35.11 (12.03)	0.15 (0.15)	0.12 (0.12)
	***p* value**	3.72e−08	0.0136	0.1397	0.1968

Remark: *SD* denotes the standard deviation. Chi-square test is used to compute the *p* values between HC and SZ groups in terms of gender. Two-sample *t*-tests are used to compute the *p* values between HC and SZ groups in terms of age and head motion.

We preprocess the rs-fMRI data using the statistical parametric mapping toolbox (SPM12). We initially exclude the first six time points, and then conduct rigid body motion correction to correct subject head motions, followed by slice-timing correction to address timing differences in slice acquisition. Next, the rs-fMRI data are transformed into the standard Montreal Neurological Institute space utilizing an echo-planar imaging template and are resampled to isotropic voxels of 3 × 3 × 3 mm^3^. The resampled rs-fMRI data then undergo smoothing with a Gaussian kernel of 6 mm full width at half maximum. Subsequently, the proposed NeuroMark pipeline (Du et al., 2020) is applied to the smoothed rs-fMRI data to obtain 53 corresponding subject-specific networks and their related time courses (TCs). Before the FNC computation, the following steps are performed on the TCs to remove noise sources, including (a) detrending linear, quadratic, and cubic trends; (b) regressing out the six head motion parameters, (c) despiking to detect and remove outliers; and (d) retaining frequencies within (0.01–0.15) Hz. Afterwards, a 53 × 53 symmetric FNC matrix is obtained by computing Pearson’s correlation coefficients between any two subject-specific functional networks for each subject. Each element in the FNC matrix is transformed to Fisher’s *Z*-score for normalization. Finally, we flatten the upper triangle of the matrix into a vector for each subject, resulting in 1,378 FNC features revealing interactions between brain networks. Notably, we carefully regress out the age, gender, and site effects prior to subsequent analyses, which minimized the influence of these effects on our final results (Du et al., 2016; Du et al., 2020).

### Different Constraint Matrices Calculated From FNC patterns

Different connectivity patterns imply distinct interaction relationships between brain functional networks, potentially reflecting disparities across different groups. In this study, we develop four different constraint matrices named ***B****^POS^*, ***B****^NEG^*, ***B****^DIF^*, and ***B****^AVG^*, based on various FNC patterns (i.e., prior knowledge). Let $D={\left\{\left(x_{i},y_{i}\right)\right\}}_{i=1}^{n}$ contains all subjects and $D^{\left(k\right)}$ includes subjects belonging to the *k*th group. Let $\mu^{\left(k\right)}=\left\{\mu_{1}^{\left(k\right)},\mu_{2}^{\left(k\right)},\ldots ,\mu_{d}^{\left(k\right)}\right\}$ represent the mean connectivity vector across subjects within $D^{\left(k\right)}$. $\varphi^{\left(k\right)}=\left\{\varphi_{1}^{\left(k\right)},\varphi_{2}^{\left(k\right)},\ldots ,\varphi_{d}^{\left(k\right)}\right\}$ represents the difference between the mean connectivity of subjects in $D^{\left(k\right)}$ and the mean connectivity of subjects in other groups (i.e., D\$D^{\left(k\right)}$). ***μ***^(*k*)^ and ***φ***^(*k*)^ are respectively denoted as

$$\mu^{\left(k\right)}=\frac{1}{n_{k}}\sum_{x_{i}\in D^{\left(k\right)}}x_{i},$$ (12)

$$\varphi^{\left(k\right)}=|\frac{1}{n_{k}}\sum_{x_{i}\in D^{\left(k\right)}}x_{i}-\frac{1}{n-n_{k}}\sum_{x_{l}\in D\backslash D^{\left(k\right)}}x_{l}|,$$ (13)

where *k* ∈ {1, …, *c*}, and*n*_*k*_ represents the number of subjects in $D^{\left(k\right)}$.

To account for the complexity of brain connectivity patterns, we employ Gaussian-form exponential functions to construct four distinct constraint matrices (***B****^POS^*, ***B****^NEG^*, ***B****^DIF^*, and ***B****^AVG^*). This design is chosen for three key reasons: (a) the inherent normalization capability of Gaussian functions naturally handles varying feature scales; (b) their smooth decay characteristics automatically regulate penalty factors based on connectivity strength; and (c) they eliminate the need for artificial hard thresholds that might inadvertently discard biologically meaningful information. Specifically, $B_{k}^{POS}$ in Equation 14 represents the *k*th column of ***B****^POS^*, which derives only from the positive mean connectivity across subjects within $D^{\left(k\right)}$, focusing on capturing abnormal FNC features associated with positive correlations between functional networks. $B_{k}^{NEG}$ in Equation 15 represents the *k*th column of ***B****^NEG^*, which derives only from the negative mean connectivity across subjects within $D^{\left(k\right)}$, focusing on capturing abnormal FNC features related to the anticorrelations between functional networks. $B_{k}^{DIF}$ in Equation 16 represents the *k*th column of ***B****^DIF^*, which derives from the mean connectivity that exhibits significant differences across subjects in $D^{\left(k\right)}$ from other groups, focusing on capturing abnormal FNC features related to variations in connectivity between groups. Lastly, $B_{k}^{AVG}$ in Equation 17 represents the *k*th column of ***B****^AVG^* and represents the mean connectivity across subjects within $D^{\left(k\right)}$, focusing on depicting abnormal FNC features related to general connectivity patterns.

$$B_{k}^{POS}=e^{-{\left(p^{\left(k\right)}/\tau \right)}^{2}},p^{\left(k\right)}=\left\{p_{1}^{\left(k\right)},p_{2}^{\left(k\right)},\ldots ,p_{d}^{\left(k\right)}\right\},\mathrm{inwhich}p_{j}^{\left(k\right)}=\max\left\{\mu_{j}^{\left(k\right)},0\right\};$$ (14)

$$B_{k}^{NEG}=e^{-{\left(q^{\left(k\right)}/\tau \right)}^{2}},q^{\left(k\right)}=\left\{q_{1}^{\left(k\right)},q_{2}^{\left(k\right)},\ldots ,q_{d}^{\left(k\right)}\right\},\mathrm{inwhich}q_{j}^{\left(k\right)}=\min\left\{\mu_{j}^{\left(k\right)},0\right\};$$ (15)

BkDIF=e−zk/τ2,zk=z1k,z2k,…,zdk,inwhichzjk=φjk,φjk≥∑j=1dφjk/d0,φjk<∑j=1dφjk/d; (16)

$$B_{k}^{AVG}=e^{-{\left(\mu^{\left(k\right)}/\tau \right)}^{2}};$$ (17)

where *j* ∈ {1, …, *d*}. ***p***^(*k*)^ is a vector that captures the positive mean connectivity across subjects in $D^{\left(k\right)}$, zeroing out all other values; ***q***^(*k*)^ is the counterpart that captures negative mean connectivity across subjects in $D^{\left(k\right)}$, zeroing out all other values; ***z***^(*k*)^ is a differential vector that captures connectivity with significant differences in the mean connectivity between subjects in $D^{\left(k\right)}$ and subjects in other groups. The parameter *τ* is empirically set to 0.2, as a comprehensive sensitivity analysis showed that varying *τ* within the range [0.1, 1] had no significant effect on the final results or conclusions. Correspondingly, we named our PriFS method using the four types of prior knowledge of ***B****^POS^*, ***B****^NEG^*, ***B****^DIF^*, and ***B****^AVG^* as PriFS^POS^, PriFS^NEG^, PriFS^DIF^, and PriFS^AVG^, respectively.

### Comparison Methods and Parameter Setting

To validate the effectiveness of the proposed method, we compare it against nine popular feature selection methods, including ReliefF, *l*_2,1_-RLDA, S^2^DFS, SIFE, ANOVA, Lasso, RFS, FS20, and ALLDA, in terms of classification performance. In subsequent ablation studies, we also compare the proposed PriFS method with the previously proposed LRFS method, which did not incorporate prior knowledge, in terms of classification performance.

Here, we set specific parameter search ranges for our PriFS method and the comparison methods in experiments. Our method searches for regularization parameters *λ*_1_, *λ*_2_, and *λ*_3_ within {10^−5^, 10^−4^, 10^−3^, 10^−2^, 10^−1^, 1, 10, 100}. Reasonably, we set parameter search ranges for each comparison method according to the corresponding literature recommendations. Specifically, ReliefF method searches for the nearest neighbor parameter within {1, 3, 5, 7, 9, 11, 13, 15}; S^2^DFS method searches for the sparse parameter within {30, 40, 50, 60, 70, 80, 90, 100, 110, 120}; SIFE method searches for population size parameter within {15, 50, 100}, with 50 iterations; Lasso method searches for regularization parameter within {1, 5, 10, 15, 20, 25, 30, 35}; ALLDA method searches for nearest neighbor parameter and generalization parameter within {1, 2, 3, 4, 5, 6} and {2, 3, 4, 5, 6, 7}, respectively; RFS method searches for regularization parameter within {10^−5^, 10^−4^, 10^−3^, 10^−2^, 10^−1^, 1, 10, 100}. It is worth noting that SIFE method outputs an optimal feature subset, while the others yield all features ranked by importance. Therefore, apart from SIFE method, the remaining comparison methods and our method consider the number of selected features (NUM) (*m*), ranging from 20 to 120 in increments of 10. ANOVA, FS20, and *l*_2,1_-RLDA methods have no other adjustable parameters.

### Experiment Procedure

We implement a rigorous ten-fold cross-validation (CV) procedure to evaluate the effectiveness of our method. Specifically, each dataset is partitioned into ten equal folds. In each iteration, nine of the ten folds (i.e., training data) are utilized for training and one fold (i.e., test data) is held out for testing. Based on the training data consisting of two diagnostic groups (i.e., HC and SZ), we first construct the constraint matrix ***B*** according to Equations 14–17. Within the training data, we perform feature selection using our proposed method. Subsequently, a classifier is trained on the selected features and its performance is evaluated on the test data. To ensure a fair and robust evaluation, we conduct an exhaustive grid search over the predefined parameter space. For each parameter combination, we repeat the ten-fold CV procedure, and the mean classification accuracy (ACC) across the ten folds is recorded. The final performance is reported using the parameter setting that yields the highest mean accuracy, which is consistent with existing work (Huang & Wu, 2021; Lim & Kim, 2021). Importantly, the same CV strategy, including parameter tuning and evaluation, is applied to all comparison methods to ensure consistency and comparability. To comprehensively evaluate the performance of the proposed method, we conduct the following experimental analyses.

To verify the discriminability and effectiveness of our PriFS method, we compare it with the nine feature selection methods using different classifiers (including SVM, 5NN, and LDA) with respect to the ACC, standard deviation (*SD*), the NUM, and runtime. In addition, we employ a pairwise two-sample *t* test with Bonferroni correction to validate the significance of the intergroup differences in classification accuracy between the proposed method and each comparison method, with a significance level of *p* = 0.05.

Additionally, we investigate the impact of the regularization parameters *λ*_1_, *λ*_2_, and *λ*_3_ on the classification performance of the proposed method to verify its robustness.

To independently assess the impact of each regularization term (i.e., constrained *l*_2,1_ sparse, graph-based, and redundancy-removal regularization) in our method on the identification of discriminative features, we perform ablation experiments. Specifically, seven variants are systematically evaluated through identical classification procedures and data partitions, combining the fitting error term with the three distinct regularization terms. These variants include: (a) ErrorG (fitting error term with graph-based regularization term); (b) ErrorR (fitting error term with redundancy-removal regularization term); (c) ErrorL (fitting error term with constrained *l*_2,1_ sparse regularization term); (d) ErrorGR (fitting error term with both graph-based and redundancy-removal regularization terms); (e) ErrorGL (fitting error term with both graph-based and constrained *l*_2,1_ sparse regularization terms); (f) ErrorRL (fitting error term with both redundancy-removal and constrained *l*_2,1_ sparse regularization terms); (g) PriFS-Pri, i.e., LRFS method, (fitting error term with graph-based, redundancy-removal, and *l*_2,1_ sparse regularization terms without prior knowledge guidance).

To evaluate the generalizability of the selected features, we conduct a cross-dataset validation experiment. Specifically, for each of the four datasets (used as training data), we perform a ten-fold CV to identify discriminative features and train the model. Features selected more than once across the ten CV folds are aggregated as the final discriminative feature subset for that dataset. The trained model is subsequently evaluated on the remaining three datasets (serving as test data) without retraining. Classification accuracies on these held-out datasets are compared to assess the cross-dataset robustness of different feature selection methods.

Furthermore, we elaborately discuss the important features identified by our method across the four datasets, emphasizing the role of different connectivity prior knowledge in identifying various features. Under the ten-fold CV procedure, we tally the frequency of each feature identified by our proposed PriFS^POS^, PriFS^NEG^, PriFS^DIF^, and PriFS^AVG^ methods across the four datasets. Additionally, by employing two-sample *t*-tests, we analyze the mean *T* values between HC and SZ groups for the selected features across the four datasets. This comprehensive analysis helps in understanding the influence of different prior knowledge in exploring critical features.

### Results and Analysis

#### Classification analysis.

**Figure 1.**
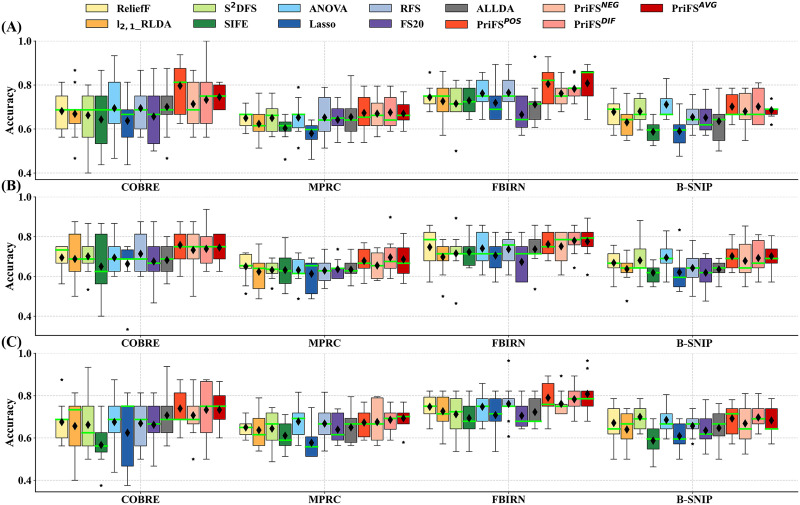
Classification accuracy obtained from nine comparison methods and the proposed methods with different prior connectivity information using (A) SVM, (B) 5NN, and (C) LDA classifiers on the four rs-fMRI datasets. In each box, the black diamond symbol “♦” represents the mean, and the green horizontal line symbol “-” represents the median. PriFS^POS^, PriFS^NEG^, PriFS^DIF^, and PriFS^AVG^ are the proposed methods using different prior connectivity information.

Employing SVM, 5NN, and LDA classifiers, we present the optimal classification results, the corresponding NUM, and runtimes for each method on the four datasets in Table 3 and Supporting Information Tables S1–S2. Figure 1 visually illustrates the classification accuracy of each method on these datasets using different classifiers. Taking the SVM classifier as an example, our method leveraging diverse connectivity information achieves optimal classification accuracy on COBRE and MPRC datasets. Although our method exhibits interfold variability on the COBRE dataset, this variability remains lower than that of most comparison methods. Furthermore, our method also outperforms most comparison methods on FBIRN and B-SNIP datasets, thanks to incorporating distinct prior knowledge. Notably, our method consistently surpasses comparison methods in mean classification performance across all datasets. Particularly, our method guided by positive connectivity information (i.e., PriFS^POS^) achieves a notable 3.89% to 11.24% performance boost over comparison methods. Overall, our method achieves superior classification performance with fewer features and comparable runtime across all datasets, demonstrating its effectiveness in identifying sparse and discriminative features with the guidance of relevant prior knowledge. It is worth mentioning that the classification results of the nine comparison methods are consistent with those reported in our previous study (Xing, Pearlson, et al., 2024), as the same data split was used within the ten-fold CV framework.

**Table 3.** Comparison of classification results for feature selection methods based on SVM classifier

		**ReliefF**	***l*_2,1_-RLDA**	**S^2^DFS**	**SIFE**	**ANOVA**	**Lasso**	**RFS**	**FS20**	**ALLDA**	**PriFS^POS^**	**PriFS^NEG^**	**PriFS^DIF^**	**PriFS^AVG^**
**COBRE**	**ACC**	0.6815	0.6688	0.6624	0.6433	0.6943	0.6369	0.6943	0.6561	0.7006	**0.7962**	**0.7134**	**0.7325**	**0.7452**
	***SD***	0.0864	0.1071	0.1190	0.1239	0.1389	0.1164	0.0958	0.1157	0.1168	0.1079	0.0963	0.1297	0.0457
	**NUM**	90	100	120	15	100	100	100	20	120	120	50	20	70
	**Time/s**	0.40	83.60	12.24	11.13	0.76	0.51	0.15	0.02	2.60	2.32	3.60	5.05	4.59
**MPRC**	**ACC**	0.6495	0.6237	0.6495	0.6031	0.6521	0.5799	0.6521	0.6418	0.6546	**0.6727**	**0.6701**	**0.6753**	**0.6701**
	***SD***	0.0451	0.0694	0.0617	0.0543	0.0740	0.0501	0.0812	0.0623	0.0805	0.0873	0.0699	0.0751	0.0507
	**NUM**	30	30	20	100	20	30	20	40	90	40	40	20	30
	**Time/s**	1.30	160.05	15.21	111.46	0.80	2.55	0.59	0.09	3.81	3.68	1.70	3.72	4.00
**FBIRN**	**ACC**	0.7438	0.7260	0.7153	0.7295	0.7616	0.7189	0.7651	0.6655	0.7117	**0.8043**	0.7616	**0.7829**	**0.8078**
	***SD***	0.0488	0.0858	0.0853	0.0579	0.0592	0.0752	0.0656	0.0652	0.0882	0.0906	0.0524	0.0447	0.0790
	**NUM**	80	50	30	50	30	120	30	30	40	40	30	20	40
	**Time/s**	0.86	142.73	12.59	76.76	0.77	1.43	0.32	0.04	2.20	4.13	5.00	3.95	4.27
**B-SNIP**	**ACC**	0.6778	0.6301	0.6802	0.5871	**0.7112**	0.5895	0.6539	0.6516	0.6348	0.7017	0.6802	0.7017	0.6850
	***SD***	0.0656	0.0618	0.0526	0.0439	0.0558	0.0655	0.0500	0.0655	0.0891	0.0581	0.0792	0.0725	0.0339
	**NUM**	60	40	50	50	40	20	70	90	100	60	20	40	40
	**Time/s**	0.56	181.39	1.72	112.71	0.80	3.97	0.67	0.10	2.55	4.87	4.37	4.41	3.85
**Mean**	**ACC**	0.6881	0.6621	0.6768	0.6408	0.7048	0.6313	0.6913	0.6537	0.6755	**0.7437**	**0.7063**	**0.7231**	**0.7270**
	***SD***	0.0615	0.0810	0.0796	0.0700	0.0820	0.0768	0.0732	0.0772	0.0937	0.0860	0.0745	0.0805	0.0523
	**NUM**	65	55	55	54	48	68	55	45	88	65	35	25	45
	**Time/s**	0.78	141.94	10.44	78.01	0.78	2.12	0.43	0.06	2.79	3.75	3.67	4.28	4.18

Remarks: ACC, *SD*, and NUM denote the mean classification accuracy, standard deviation, and the number of selected features, respectively. The underlined and **bold** text indicates that the method achieves optimal accuracy. PriFS^POS^, PriFS^NEG^, PriFS^DIF^, and PriFS^AVG^ are the proposed methods using different prior connectivity information.

Taking the SVM classifier as an example, we conduct a significance analysis on the classification accuracy of the proposed method compared with the nine comparison methods, as shown in Table 4 and Supporting Information Tables S3–S5. On the COBRE and FBIRN datasets, our PriFS^POS^ method demonstrates outstanding classification performance, significantly outperforming the nine comparison methods (*p* ≤ 0.05 with Bonferroni correction); likewise, on the MPRC and B-SNIP datasets, PriFS^POS^ method also significantly surpasses eight of the comparison methods, as detailed in Table 4. Similarly, the PriFS^NEG^, PriFS^DIF^, and PriFS^AVG^ methods also significantly surpass most of the comparison methods in classification performance on the four datasets (*p* ≤ 0.05 with Bonferroni correction), as shown in Supporting Information Tables S3–S5. In conclusion, our method demonstrates statistically significant superiority over most comparison methods across all four datasets (*p* ≤ 0.05 with Bonferroni correction). These experimental results further validate the significant advantage of the proposed method in identifying discriminative features.

**Table 4.** Significance analysis of classification accuracy between the proposed PriFS^POS^ method and each comparison method based on SVM classifier

		**ReliefF**	***l*_2,1_-RLDA**	**S^2^DFS**	**SIFE**	**ANOVA**	**Lasso**	**RFS**	**FS20**	**ALLDA**
**COBRE**	***p* value**	4.26e−24	2.73e−23	2.12e−28	2.25e−40	9.14e−14	4.86e−29	6.83e−16	2.10e−33	5.61e−14
	***T* value**	12.07	11.77	13.64	18.18	8.18	13.88	9.01	15.50	8.27
**MPRC**	***p* value**	6.63e−11	7.56e−24	9.15e−09	3.87e−35	7.98e−11	1.38e−75	6.61e−12	1.65e−06	1.76e−04
	***T* value**	6.72	10.77	5.87	13.71	6.69	23.28	7.09	4.87	3.79
**FBIRN**	***p* value**	2.03e−24	7.76e−32	2.41e−24	2.40e−20	8.47e−10	9.21e−32	1.92e−08	8.12e−58	1.95e−44
	***T* value**	11.23	13.36	11.21	10.00	6.35	13.34	5.79	20.54	16.84
**B-SNIP**	***p* value**	1.70e−07	5.78e−69	1.15e−15	1.43e−146	1.09e−02	5.75e−86	3.78e−34	2.11e−76	1.52e−46
	***T* value**	5.32	21.36	8.33	40.44	−2.56	25.22	13.36	23.04	16.29

Remarks: Underlined text indicates that the PriFS^POS^ method significantly outperforms the comparison methods in terms of classification accuracy (*p* ≤ 0.05 with Bonferroni correction).

#### Parameter sensitivity analysis.

**Figure 2.**
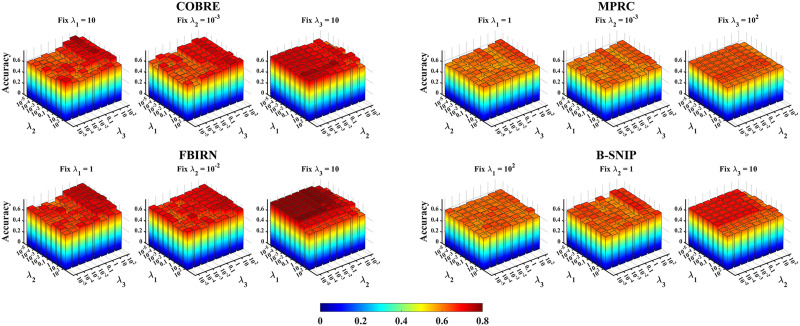
The parameter sensitivity analysis diagram of the proposed method using positivity connectivity information on the four datasets. *λ*_1_, *λ*_2_, and *λ*_3_ are the regularization parameters for the graph-based regularization term, redundancy-removal regularization term, and constrained *l*_2,1_ sparse regularization term, respectively, which control the tradeoff among these terms.

In this section, we discuss the influences of the involved regularization parameters *λ*_1_, *λ*_2_, and *λ*_3_ on the ACC. Focusing on the PriFS^POS^ method due to space limitations, as shown in Figure 2, we analyze the impact of different *λ*_1_, *λ*_2_, and *λ*_3_ on ACC using the four datasets. It becomes evident that, when *λ*_3_ is fixed, the change in ACC is marginal as parameters *λ*_1_ and *λ*_2_ vary. With either *λ*_1_ or *λ*_2_ fixed, the corresponding ACC tends to be generally high when *λ*_3_ equals to 10. Overall, even though optimal parameters may vary across different datasets, our method exhibits relatively stable classification performance, highlighting its insensitivity to parameter variations.

#### Ablation analysis.

In this section, we evaluate the impact of each regularization term in the objective function of Equation 5 on identifying discriminative features through ablation experiments. Due to space limitations, we present the classification results of the PriFS^POS^ method and its seven variants in Table 5. It is evident that PriFS^POS^ outperforms all variants across all four datasets. The absence of any of the three regularization terms results in reduced classification performance, suggesting that PriFS^POS^ actually benefits from the synergistic combination of the three regularization terms. Particularly, compared with PriFS-Pri method, PriFS^POS^ significantly improves classification performance, emphasizing the importance of the incorporation of prior knowledge. By integrating the results from Table 5 and Table 3, we observe that PriFS^DIF^ and PriFS^AVG^ also achieve higher ACC across all four datasets compared with PriFS-Pri. In conclusion, we can infer that the three regularization terms collectively contribute to the improved performance of the proposed method.

**Table 5.** Results for the ablation experiments of the proposed method using positivity connectivity information based on SVM classifier

		**ErrorG**	**ErrorR**	**ErrorL**	**ErrorGR**	**ErrorGL**	**ErrorRL**	**PriFS-Pri**	**PriFS^POS^**
**COBRE**	**ACC**	0.6752	0.6624	0.7325	0.7197	0.7771	0.7325	0.7452	**0.7962**
	***SD***	0.1043	0.1078	0.0878	0.0917	0.0945	0.0878	0.1404	0.1079
	**NUM**	120	120	110	30	110	110	40	120
	**Time/s**	0.14	0.08	1.04	0.09	4.34	1.05	1.61	2.32
**MPRC**	**ACC**	0.6366	0.6314	0.6418	0.6469	0.6598	0.6649	**0.6727**	**0.6727**
	***SD***	0.0473	0.0844	0.0903	0.0769	0.0487	0.0486	0.0667	0.0873
	**NUM**	30	50	50	40	20	50	20	40
	**Time/s**	0.05	0.06	1.05	0.10	3.65	4.29	1.89	3.68
**FBIRN**	**ACC**	0.7189	0.6868	0.7936	0.7367	0.7972	0.8007	0.7651	**0.8043**
	***SD***	0.0906	0.0800	0.0706	0.0620	0.0749	0.0840	0.0538	0.0906
	**NUM**	120	50	30	120	40	30	110	40
	**Time/s**	0.03	0.08	1.24	0.04	10.26	1.96	4.85	4.13
**B-SNIP**	**ACC**	0.6635	0.6372	0.6850	0.6468	0.6969	0.6921	0.6921	**0.7017**
	***SD***	0.0633	0.0852	0.0826	0.0757	0.0669	0.0713	0.0627	0.0581
	**NUM**	70	50	50	40	60	50	30	60
	**Time/s**	0.04	0.10	1.09	0.08	4.22	1.08	2.01	4.87

Remarks: ACC, *SD*, and NUM denote classification accuracy, standard deviation, and the number of selected features, respectively. The underlined and **bold** text indicates that the method achieves optimal accuracy.

#### Cross-dataset validation.

As demonstrated in Table 6, the cross-dataset validation results reveal the superiority of the proposed method in generalization performance compared with comparison methods. Specifically, the PriFS^POS^ method achieves the highest ACC of 0.6706, followed by the PriFS^DIF^ method with 0.6630 accuracy, both outperforming all nine comparison methods. Furthermore, the PriFS^AVG^ method surpasses seven out of nine comparison methods. These results substantiate that our method, particularly the PriFS^POS^, PriFS^DIF^, and PriFS^AVG^, exhibits exceptional cross-dataset generalizability. These findings collectively demonstrate that our method identifies neurobiologically stable features with preserved discriminability across independent cohorts, while the discovered FNC features exhibit both abnormality-specificity and cross-subject reliability.

**Table 6.** Classification accuracy through cross-dataset validation

		**ReliefF**	***l*_2,1_-RLDA**	**S^2^DFS**	**SIFE**	**ANOVA**	**Lasso**	**RFS**	**FS20**	**ALLDA**	**PriFS^POS^**	**PriFS^NEG^**	**PriFS^DIF^**	**PriFS^AVG^**
**COBRE**	**FBIRN**	0.6548	0.6441	0.6584	0.6228	0.6619	0.6050	0.6512	0.5872	0.5907	**0.6748**	0.6512	0.6157	0.6157
	**B-SNIP**	0.6516	0.6158	0.5943	0.6134	0.5943	0.6301	0.5990	0.6014	0.5895	**0.6611**	0.6110	0.5967	**0.6348**
	**MPRC**	0.6314	0.6211	0.6108	0.6392	0.6211	0.6314	0.6057	0.6031	0.6340	**0.6418**	0.6057	0.6186	**0.6418**
**MPRC**	**FBIRN**	0.7011	0.6228	0.6726	0.6335	0.6797	0.6868	0.6797	**0.7082**	0.6228	0.6748	0.6904	0.7011	0.6477
	**B-SNIP**	0.6253	0.6444	0.6253	0.6420	0.6181	0.6492	0.6181	0.6348	0.5871	**0.6611**	**0.6730**	0.6301	0.5871
	**COBRE**	**0.7006**	0.6561	0.6306	0.6879	0.6752	0.6752	0.6752	0.6943	0.5669	0.6906	0.6051	**0.7006**	0.6369
**FBIRN**	**B-SNIP**	0.6444	0.6038	0.6062	0.6134	0.6038	0.6086	0.5823	0.5823	0.5823	**0.6496**	0.6014	**0.6563**	0.6420
	**MPRC**	0.6675	0.6314	0.6108	0.6237	0.6392	0.6495	0.6366	0.6186	0.6160	0.6592	0.6082	**0.6804**	0.6546
	**COBRE**	0.6561	0.6879	0.6452	0.6051	0.6752	0.5924	0.6879	0.6306	0.5669	0.6569	0.6115	0.6688	**0.6943**
**B-SNIP**	**FBIRN**	0.6619	0.6121	0.7117	0.6868	0.7189	0.6548	0.6904	0.6335	0.6121	0.6897	0.6299	**0.7616**	**0.7331**
	**MPRC**	0.6418	0.6443	0.6727	0.6598	0.6778	0.6314	0.6469	0.6598	0.6546	**0.6933**	0.6521	**0.6830**	0.6521
	**COBRE**	0.6178	0.6752	0.6624	0.6815	0.6879	0.5541	0.6815	0.6497	0.6879	**0.6943**	0.5860	0.6433	0.6815
**Mean**		0.6545	0.6382	0.6418	0.6424	0.6544	0.6307	0.6462	0.6336	0.6092	**0.6706**	0.6271	**0.6630**	0.6518

#### Selected FNC features and analysis.

**Figure 3.**
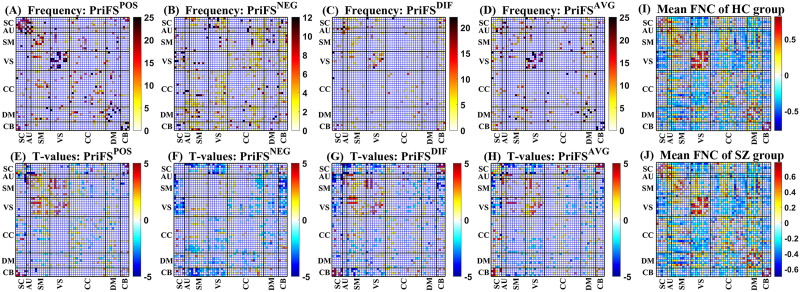
Heatmaps of functional network connectivity features across the four datasets. (A–D) Frequency for features selected by PriFS^POS^, PriFS^NEG^, PriFS^DIF^, and PriFS^AVG^ methods, respectively. (E–H) *T* values between HC and SZ groups for features selected by PriFS^POS^, PriFS^NEG^, PriFS^DIF^, and PriFS^AVG^ methods, respectively. (I) Mean FNC matrix across all subjects in HC group of the four datasets. (J) Mean FNC matrix across all subjects in SZ group of the four datasets. Brain is divided into seven functional domains, including SC, AU, SM, VS, cognitive-control (CC), default-mode (DM), and CB domains.

In this section, we delve into a detailed analysis of the differences between HC group and SZ group in FNC features identified by the proposed method with different prior knowledge. Figure 3A–D depict the frequency of selected features by PriFS^POS^, PriFS^NEG^, PriFS^DIF^, and PriFS^AVG^ across the four datasets, respectively. Figure 3I–J displays the mean FNCs across subjects of HC group and SZ group in the four datasets, respectively. Notably, the features selected by PriFS^POS^ and PriFS^NEG^ are remarkably different. The former tends to select more internal positive connectivity within brain domains, such as subcortical (SC), auditory (AU), visual (VS), and cerebellar (CB) domains, while the latter leans toward selecting negative connectivity between brain domains, notably between SC and VS domains. Features selected by PriFS^DIF^ and PriFS^AVG^ appear to represent a blend of the characteristics favored by PriFS^POS^ and PriFS^NEG^, including both internal- and inter-domain connectivity.

Figure 3E–H illustrates the mean *T* values between HC and SZ groups of the FNC features selected by PriFS^POS^, PriFS^NEG^, PriFS^DIF^, and PriFS^AVG^ across the four datasets, respectively. PriFS^POS^ tends to select features with *T* values greater than 0, while PriFS^NEG^ selects features with *T* values less than 0. Similarly, PriFS^DIF^ and PriFS^AVG^ select features with both positive and negative *T* values. Overall, there is a tendency for positive *T* values within the VS, AU, and CB domains. Regarding inter-domain connectivity, positive *T* values are observed between SC and CB as well as between sensorimotor (SM) and VS domains, while negative *T* values are noted between SC and VS, SM and CB, as well as between VS and CB domains.

## DISCUSSION

**Figure 4.**
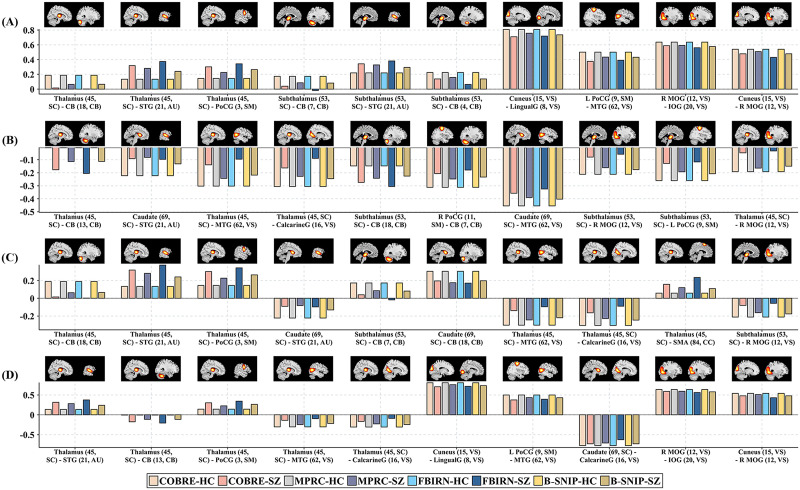
The mean connectivity strength of the top 10 high-frequency features selected by our proposed method within each group in each dataset. (A–D) High-frequency features selected by PriFS^POS^, PriFS^NEG^, PriFS^DIF^, and PriFS^AVG^ methods, respectively. Brain is divided into seven functional domains, including SC, AU, SM, VS, CC, DM, and CB domains. CalcarineG: calcarine gyrus; CB: cerebellum; IOG: inferior occipital gyrus; LingualG: lingual gyrus; MTG: middle temporal gyrus; R MOG: right middle occipital gyrus; PoCG: postcentral gyrus; L PoCG: left postcentral gyrus; R PoCG: right postcentral gyrus; SMA: supplementary motor area; STG: superior temporal gyrus. The red highlighted areas in the brain above the bar represent the corresponding functional network. The figure in parentheses represents the corresponding functional network ID.

In this section, we engage in a thorough discussion of the top 10 high-frequency important FNC features that are most commonly selected across different datasets by our method using each of the four types of prior knowledge. Figure 4A–D displays the mean connectivity strength values within each group of the top 10 high-frequency features identified by PriFS^POS^, PriFS^NEG^, PriFS^DIF^, and PriFS^AVG^ for each dataset, respectively. It is evident that PriFS^POS^ tends to select positively correlated connectivity between functional networks, exhibiting decreased connectivity in seven out of the 10 features in the SZ group compared with the HC group. Conversely, PriFS^NEG^ favors negatively correlated connectivity between functional networks, with eight out of the 10 features exhibiting reduced amplitude of connectivity in the SZ group compared with the HC group. PriFS^DIF^ and PriFS^AVG^, on the other hand, select features with both positively and negatively correlated connectivity between functional networks, with the majority capturing reduced connectivity amplitudes in SZ group relative to HC group. Supporting Information Tables S6–S10 provide a comprehensive overview, including the mean connectivity strength values, *p* values, *T* values of HC and SZ groups, and changes of connectivity amplitude in SZ group, for these features selected by our method on each dataset. The multifaceted results obtained using different prior knowledge all point to impaired intra- and internetwork interaction in SZ.

Our findings demonstrate that leveraging distinct prior information enables the proposed method to uncover functional connectivity abnormalities in SZ from complementary perspectives. Importantly, these results not only align with some prior findings of SZ but also reveal novel patterns of abnormalities that refine current understanding. Specifically, significant alterations occur in positively correlated connectivity within the SZ group compared with the HC group. Our PriFS^POS^ method reveals enhanced connectivity between thalamus/subthalamus and superior temporal gyrus (STG) as well as between thalamus and postcentral gyrus (PoCG) in the SZ group. Additionally, there is reduced connectivity between the cuneus and lingual gyrus (LingualG), cuneus and right middle occipital gyrus (R MOG), R MOG and inferior occipital gyrus (IOG), and left PoCG (L PoCG) and middle temporal gyrus (MTG) within the SZ group. Conversely, there are also notable aberrations in the negatively correlated connectivity in the SZ group. Our PriFS^NEG^ method identifies increased negative correlations concerning thalamus/subthalamus connectivity with the cerebellum in the SZ group. Additionally, diminished negative correlations are also observed in caudate-STG connectivity, caudate-MTG connectivity, thalamus-MTG connectivity, thalamus-calcarine gyrus (CalcarineG) connectivity, thalamus/subthalamus-R MOG connectivity, subthalamus-L PoCG connectivity, and right PoCG-cerebellum connectivity in the SZ group. Moreover, our PriFS^DIF^ method uniquely captures FNC with significant differences in mean connectivity strengths between the SZ group and the HC group. This includes a decreased positive correlation between the caudate and cerebellum, as well as an enhanced positive correlation between the thalamus and the supplementary motor area (SMA) in the SZ group. PriFS^AVG^ uniquely reveals a decreased negative correlation between the caudate and CalcarineG.

The diminished positively correlated connectivity within the VS domain (i.e., LingualG, cuneus, and R MOG) provides direct neurobiological correlates for visual perception disturbances in SZ. These occipital regions, critical for visual information processing, exhibit functional decoupling that may contribute to visual hallucinations and distortions in SZ (M. Cai et al., 2022). Furthermore, weakened positive caudate-cerebellum connectivity reflects abnormal regulation of the striatum-cerebellum circuit, potentially linking to higher-order executive function and working memory deficits (Acharya, Ren, Yi, Tian, & Liang, 2023). Conversely, enhanced thalamic positive connectivity reveals divergent pathological pathways: thalamus-STG hyperconnectivity may drive auditory hallucinations via dysregulated thalamocortical auditory circuits (Xing, van Erp, et al., 2024), while thalamus-SMA (a part of motor cortices) hyperconnectivity suggests aberrant sensorimotor integration, potentially underlying motor abnormalities (Walther et al., 2017). Overall, our PriFS^POS^ method specifically highlights abnormalities within positively correlated connectivity, reflecting impaired functional cooperation between brain networks.

Notably, we observe a novel dysregulation pattern characterized by weakened negative correlations between the SC and VS domains. Unlike prior reports of broad subcortical-visual abnormalities (Butler et al., 2007), our findings specifically demonstrate disrupted inhibitory interactions between these domains, offering more precise mechanistic insights. Specifically, the reduced negative correlations between the caudate and the MTG/CalcarineG, as well as between the thalamus and the MTG/CalcarineG/R MOG, may reflect impaired inhibitory control over associative visual cortices, potentially contributing to perceptual distortions or hallucinations. In addition, the discovery of diminished postcentral gyrus-cerebellum negative correlation represents a previously unreported abnormality with mechanistic implications. Disruptions between the PoCG (involved in somatosensory processing) (DiGuiseppi & Tadi, 2023) and the cerebellum (involved in motor control and cognitive functions) (Carey, 2024) may reflect impaired inhibition between sensorimotor and higher-order regulatory systems, potentially contributing to motor coordination deficits, hallucinations, and cognitive impairments in SZ.

More importantly, our method enables to disentangle directionally distinct thalamus-cerebellum connectivity changes. Guided by distinct prior information, our method collectively reveals a more refined bidirectional dysregulation pattern, characterized by the coexistence of reduced positive correlations and enhanced negative correlations. These findings suggest divergent pathological mechanisms: the reduction in positive correlations may reflect impaired cerebellar modulation of thalamic activity, leading to deficits in thalamic integration and associated symptoms such as delusions and cognitive impairments; whereas the enhancement of negative correlations may indicate pathological inhibitory regulation between the thalamus and cerebellum, potentially contributing to motor coordination dysfunction. Such differential alterations were previously obscured by conventional connectivity analyses that conflate correlation directions.

Furthermore, our PriFS method guided by different prior knowledge identifies important features with varying discriminability in distinguishing between the HC and SZ groups. Compared with our PriFS method that utilizes the remaining three types of prior knowledge, PriFS^NEG^ method that only incorporates negatively correlated prior knowledge demonstrates a slightly lower capability to identify discriminative features for SZ. In short, our method flexibly incorporates various prior knowledge to guide the selection of discriminative and target-relevant features, such as focusing on the identification of abnormalities in SZ group between functional networks exhibiting mutual synergy or inhibition. This nuanced perspective not only adds depth to our understanding of the dysfunction in SZ group but also highlights the specificity of certain connectivity patterns, underscoring the value of integrating various prior knowledge to uncover specific, target-oriented, and significant neural biomarkers.

## CONCLUSION

In this paper, we propose a PriFS method to explore specific, target-oriented, and critical biomarkers for psychiatric disorders. Our method constructs a constrained *l*_2,1_ sparse regularization term to incorporate brain-specific prior knowledge, thereby enhancing the interpretability of the identified features. Additionally, we integrate a graph-based regularization term and a redundancy-removal term to improve feature discriminability and minimize redundancy, respectively. Experiments on the rs-fMRI datasets demonstrate the superior performance of our PriFS method over nine state-of-the-art feature selection methods in accurately identifying discriminative FNC features for distinguishing SZ patients from HCs. Moreover, the FNC features identified by our method provide specific insights into the pathogenesis of SZ, offering the potential for more accurate diagnosis strategies in psychiatry.

Our study has three limitations. First, while our method demonstrates robustness across parameter ranges, optimal performance requires manual adjustment of three regularization parameters. Second, while our method is theoretically generalizable across disorders and data modalities, current validation has focused solely on connectivity features in SZ. Third, the observed FNC abnormalities may reflect combined disease-specific and treatment-related effects, as medication confounds were not explicitly regressed out. Future work will (a) develop automated parameter optimization; (b) validate the framework across multimodal data, diverse psychiatric disorders, and comorbid populations; and (c) disentangle disease-specific neural signatures from treatment effects.

## ACKNOWLEDGMENTS

The work was supported by the National Natural Science Foundation of China (62576199 and 62076157); Sanjin Talent Science and Technology Innovation Leading Talent Project; and National Institutes of Health (R01MH123610). The FBIRN data collection was supported by the National Institutes of Health (1U24RR021992).

## SUPPORTING INFORMATION

Supporting information for this article is available at https://doi.org/10.1162/NETN.a.37.

## AUTHOR CONTRIBUTIONS

Ying Xing: Conceptualization; Formal analysis; Investigation; Methodology; Software; Validation; Visualization; Writing – original draft; Writing – review & editing. Godfrey D. Pearlson: Data curation; Writing – review & editing. Peter Kochunov: Data curation; Writing – review & editing. Vince D. Calhoun: Data curation; Funding acquisition; Writing – review & editing. Yuhui Du: Conceptualization; Data curation; Funding acquisition; Investigation; Methodology; Project administration; Supervision; Writing – original draft; Writing – review & editing.

## FUNDING INFORMATION

Yuhui Du, National Natural Science Foundation of China, Award ID: 62576199. Yuhui Du, National Natural Science Foundation of China, Award ID: 62076157. Yuhui Du, Sanjin Talent Science and Technology Innovation Leading Talent Project. Vince D. Calhoun, National Institutes of Health, Award ID: R01MH123610. National Institutes of Health, Award ID: 1U24RR021992.

## DATA AND CODE AVAILABILITY

The COBRE and B-SNIP datasets utilized in this study are sourced from publicly available databases, found at https://fcon_1000.projects.nitrc.org/indi/retro/cobre.html and https://nda.nih.gov, respectively. The FBIRN and MPRC datasets are not publicly available due to privacy or ethical restrictions. The reference numbers of the institutional review board approvals for the FBIRN and MPRC data are HS No. 2009-7128 and HP-00045716, respectively. The datasets used in the analyses are completely de-identified.

The code will be made available on request.

## Supplementary Material



## TECHNICAL TERMS

Biomarker: Objective and reliable biological measures indicating disease mechanisms and aiding diagnosis.
Feature selection: The process of selecting important and relevant features from high-dimensional data.
Prior knowledge: Preexisting validated information used to guide analysis.
Graph-based regularization term: A constraint preserving data local structure to enhance feature discriminability.
Redundancy-removal term: A constraint minimizing inter-feature similarity to ensure independence between selected features.
Functional network connectivity: Statistical dependencies between time series of brain networks to reflect functional interactions.
Constrained *l*_2,1_ sparse regularization term: A constraint enforcing feature sparsity while prioritizing target-specific connectivity features.
NeuroMark: An automated and adaptive independent component analysis pipeline for deriving fMRI-based brain network measures.
Functional cooperation: Positive connectivity between brain networks enabling collaborative information processing.
Bidirectional dysregulation: Concurrent abnormalities of both synergistic and inhibitory connectivity patterns.
