# Supplementary material for: A prior-knowledge-guided feature selection method and its application to biomarker identification of schizophrenia
Source: Netw Neurosci. 2025 Nov 20;9(4):1423–48. doi: 10.1162/NETN.a.37 (PMC12635834; doi:10.1162/NETN.a.37)
Supplement: Supplementary file 1 [file netn-9-4-1423-s001.pdf]

Supplementary Materials

Supplementary Proofs

Here, we provide the justification that the objective function in Eq. (5) possesses a global optimal solution. Let $J(\boldsymbol{W})$ represent the objective function in Eq. (5). The fitting error term, graph-based regularization (i.e., Eq. (2)), redundancy-removal regularization (i.e., Eq. (3)), and constrained $l_{2,1}$ sparse regularization (i.e., Eq. (1)) terms in $J(\boldsymbol{W})$ are all convex with respect to $\boldsymbol{W}$, thereby ensuring the existence of a global optimal solution. Next, we will demonstrate that $J(\boldsymbol{W})$ does not increase with the iteration in Algorithm 1, i.e., $J(\boldsymbol{W}^{(t+1)}) \leq J(\boldsymbol{W}^{(t)})$, by proving the following two Lemmas.

Lemma 1: $F(\boldsymbol{W})$ is an auxiliary function defined as

$$F(\boldsymbol{W}) = ||\boldsymbol{Y} - \boldsymbol{X}\boldsymbol{W}||_F^2 + \lambda_1 Tr(\boldsymbol{W}^T\boldsymbol{X}^T\boldsymbol{L}\boldsymbol{X}\boldsymbol{W}) + \lambda_2 Tr(\boldsymbol{W}^T\boldsymbol{Q}\boldsymbol{W}) + \lambda_3\left(\sum_{i=1}^{d}\frac{\sum_{j=1}^{c}(B_{ij}W_{ij})^2}{2\sqrt{\sum_{k=1}^{c}\left(B_{ik}W_{ik}{}^{(t)}\right)^2}}\right). \quad (A.1)$$

Along with the iteration progresses in Algorithm 1, the following inequality holds,

$$F\left(\boldsymbol{W}^{(t+1)}\right) \leq F\left(\boldsymbol{W}^{(t)}\right). \quad (A.2)$$

Proof. By differentiating $F(\boldsymbol{W})$ with respect to W_{ij}, we get

$$\frac{\partial F(\boldsymbol{W})}{\partial W_{ij}} = 2(\boldsymbol{X}^T\boldsymbol{X}\boldsymbol{W} - \boldsymbol{X}^T\boldsymbol{Y})_{ij} + 2\lambda_1(\boldsymbol{X}^T\boldsymbol{L}\boldsymbol{X}\boldsymbol{W})_{ij} + 2\lambda_2(\boldsymbol{Q}\boldsymbol{W})_{ij} + \lambda_3\frac{(\boldsymbol{B}\odot\boldsymbol{B}\odot\boldsymbol{W})_{ij}}{\sqrt{\sum_{k=1}^{c}\left(B_{ik}W_{ik}{}^{(t)}\right)^2}}. \quad (A.3)$$

Let diagonal matrix

$$\boldsymbol{U}^{(t)} = diag\left((\sum_{k=1}^{c}\left(B_{1k}W_{1k}^{(t)}\right)^2)^{-\frac{1}{2}}, \dots, (\sum_{k=1}^{c}\left(B_{dk}W_{dk}^{(t)}\right)^2)^{-\frac{1}{2}}\right), \quad (A.4)$$

Then,

$$\frac{\partial F(\boldsymbol{W})}{\partial \boldsymbol{W}} = (2\boldsymbol{X}^T\boldsymbol{X}\boldsymbol{W} - 2\boldsymbol{X}^T\boldsymbol{Y}) + 2\lambda_1\boldsymbol{X}^T\boldsymbol{L}\boldsymbol{X}\boldsymbol{W} + 2\lambda_2\boldsymbol{Q}\boldsymbol{W} + \lambda_3\boldsymbol{U}^{(t)}(\boldsymbol{B}\odot\boldsymbol{B}\odot\boldsymbol{W}). \quad (A.5)$$

Let $\frac{\partial F(\boldsymbol{W})}{\partial \boldsymbol{W}} = 0$, then,

$$(\boldsymbol{X}^T\boldsymbol{X}\boldsymbol{W}) + \lambda_1\boldsymbol{X}^T\boldsymbol{L}\boldsymbol{X}\boldsymbol{W} + \lambda_2\boldsymbol{Q}\boldsymbol{W} + \frac{\lambda_3}{2}\boldsymbol{U}^{(t)}(\boldsymbol{B}\odot\boldsymbol{B}\odot\boldsymbol{W}) = \boldsymbol{X}^T\boldsymbol{Y}. \quad (A.6)$$

It is difficult to solve for $\boldsymbol{W}$ as a whole. Therefore, we seek the optimal $\boldsymbol{W}^*$ by solving for each column in $\boldsymbol{W}$, resulting in $\boldsymbol{W}^* = [\boldsymbol{W}_1{}^*, \boldsymbol{W}_2{}^*, \dots, \boldsymbol{W}_c{}^*]$. For the jth column $\boldsymbol{W}_j$ in $\boldsymbol{W}$:

$$\boldsymbol{X}^T\boldsymbol{X}\boldsymbol{W}_j + \lambda_1\boldsymbol{X}^T\boldsymbol{L}\boldsymbol{X}\boldsymbol{W}_j + \lambda_2\boldsymbol{Q}\boldsymbol{W}_j + \frac{\lambda_3}{2}\boldsymbol{U}^{(t)}\left(\boldsymbol{B}_j\odot\boldsymbol{B}_j\odot\boldsymbol{W}_j\right) = \boldsymbol{X}^T\boldsymbol{Y}_j. \quad (A.7)$$

Let diagonal matrix

$$\boldsymbol{M} = diag\left\{\left(\frac{\lambda_3}{2}U_{11}^{(t)}\right)B_{1j}{}^2, \ldots, \left(\frac{\lambda_3}{2}U_{dd}^{(t)}\right)B_{dj}{}^2\right\}. \tag{A.8}$$

Then,

$$\boldsymbol{W}_j{}^* = (\boldsymbol{X}^T\boldsymbol{X} + \lambda_1\boldsymbol{X}^T\boldsymbol{L}\boldsymbol{X} + \lambda_2\boldsymbol{Q} + \boldsymbol{M})^{-1}(\boldsymbol{X}^T\boldsymbol{Y}_j). \tag{A.9}$$

Through numerous iterations, we can derive the optimal solution $\boldsymbol{W}^* = [\boldsymbol{W}_1{}^*, \ldots, \boldsymbol{W}_c{}^*]$, for the auxiliary function in Eq. (A.1). This implies that for any $\boldsymbol{W}$, the inequality $F(\boldsymbol{W}^*) \le F(\boldsymbol{W})$ holds true, subsequently indicating $F(\boldsymbol{W}^{(t+1)}) \le F(\boldsymbol{W}^{(t)})$. The proof of Lemma 1 is completed.

Lemma 2: With each iteration of Algorithm 1, the transformation matrix $\boldsymbol{W}$ exhibits the following characteristic

$$J(\boldsymbol{W}^{(t+1)}) - J(\boldsymbol{W}^{(t)}) \le F(\boldsymbol{W}^{(t+1)}) - F(\boldsymbol{W}^{(t)}). \tag{A.10}$$

Proof.

$$\Delta = (J(\boldsymbol{W}^{(t+1)}) - J(\boldsymbol{W}^{(t)})) - (F(\boldsymbol{W}^{(t+1)}) - F(\boldsymbol{W}^{(t)})). \tag{A.11}$$

By substituting Eq. (A.1) and Eq. (5) into Eq. (A.11), we can obtain

$$\Delta = \lambda_3\sum_{i=1}^{d}\sum_{j=1}^{c}\left(B_{ij}W_{ij}{}^{(t+1)}\right)^2 - \lambda_3\sum_{i=1}^{d}\sum_{j=1}^{c}\left(B_{ij}W_{ij}{}^{(t)}\right)^2 - \lambda_3\left(\sum_{i=1}^{d}\frac{\sum_{j=1}^{c}\left(B_{ij}W_{ij}{}^{(t+1)}\right)^2}{2\sqrt{\sum_{j=1}^{c}\left(B_{ij}W_{ij}{}^{(t)}\right)^2}}\right) + \lambda_3\left(\sum_{i=1}^{d}\frac{\sum_{j=1}^{c}\left(B_{ij}W_{ij}{}^{(t)}\right)^2}{2\sqrt{\sum_{j=1}^{c}\left(B_{ij}W_{ij}{}^{(t)}\right)^2}}\right)$$

$$= -\lambda_3\sum_{i=1}^{d}\left(\left(\sum_{j=1}^{c}\left(\boldsymbol{B}_{ij}\boldsymbol{W}_{ij}{}^{(t)}\right)^2\right)^{-\frac{1}{2}}\left(\left(\sum_{j=1}^{c}\left(\boldsymbol{B}_{ij}\boldsymbol{W}_{ij}{}^{(t+1)}\right)^2\right)^{\frac{1}{2}} - \left(\sum_{j=1}^{c}\left(\boldsymbol{B}_{ij}\boldsymbol{W}_{ij}{}^{(t)}\right)^2\right)^{\frac{1}{2}}\right)^2\right) \le 0 \tag{A.12}$$

Thus, $J(\boldsymbol{W}^{(t+1)}) - J(\boldsymbol{W}^{(t)}) \le F(\boldsymbol{W}^{(t+1)}) - F(\boldsymbol{W}^{(t)}) \le 0$. The proof of Lemma 2 is completed.

Consequently, in accordance with Lemma 1 and Lemma 2, the inequality $J(\boldsymbol{W}^{(t+1)}) \le J(\boldsymbol{W}^{(t)})$ holds. This implies that during the iterative process of Algorithm 1, the value of $J(\boldsymbol{W})$ does not increase, thereby demonstrating that Algorithm 1 exhibits monotonic convergence.

Supplementary Tables

Table S1. Comparison of classification results for feature selection methods based on 5NN classifier

		ReliefF	$l_{2,1}$-RLDA	S^2DFS	SIFE	ANOVA	Lasso	RFS	FS20	ALLDA	PriFS^{POS}	PriFS^{NEG}	PriFS^{DIF}	PriFS^{AVG}
COBRE	ACC	0.6943	0.6879	0.7006	0.6497	0.6943	0.6624	0.7134	0.6752	0.6815	**0.7580**	**0.7325**	**0.7389**	**0.7452**
	SD	0.0620	0.1172	0.0874	0.1358	0.0855	0.1124	0.0981	0.1290	0.0669	0.0803	0.1044	0.0918	0.0642
	NUM	60	40	60	166	70	110	60	110	120	70	50	80	90
	Time/s	0.4104	83.6036	12.2380	11.1319	0.7575	0.4444	0.1533	0.0224	2.6034	2.9964	4.4170	4.1588	4.3009
MPRC	ACC	0.6495	0.6237	0.6314	0.6314	0.6314	0.6134	0.6289	0.6366	0.6340	**0.6778**	**0.6546**	**0.6959**	**0.6856**
	SD	0.0660	0.0819	0.0435	0.0870	0.0636	0.0795	0.0593	0.0439	0.0511	0.0611	0.0644	0.0858	0.0809
	NUM	90	60	70	36	20	100	50	90	90	50	30	50	60
	Time/s	0.4834	160.0539	15.2101	111.4586	0.8027	2.6581	0.5878	0.0861	2.9325	3.8835	4.0354	4.2881	4.1324
FBIRN	ACC	0.7473	0.6975	0.7153	0.7224	0.7402	0.7046	0.7367	0.6726	0.7367	**0.7616**	**0.7509**	**0.7794**	**0.7758**
	SD	0.0859	0.0810	0.1011	0.0622	0.0759	0.0758	0.0625	0.0934	0.0842	0.0592	0.0753	0.0590	0.0734
	NUM	80	40	100	64	50	90	50	40	120	120	50	100	80
	Time/s	0.8029	142.7261	12.5939	152.7448	0.7699	1.3771	0.3170	0.0440	2.3089	4.1681	4.8042	4.2711	4.2959
B-SNIP	ACC	0.6683	0.6372	0.6802	0.6181	0.6945	0.6205	0.6420	0.6181	0.6348	**0.7017**	0.6778	0.6921	**0.7017**
	SD	0.0558	0.0645	0.0966	0.0453	0.0695	0.0924	0.0718	0.0806	0.0394	0.0588	0.0857	0.0809	0.0659
	NUM	110	110	110	199	110	90	120	80	100	100	70	30	120
	Time/s	1.4160	181.3869	1.7219	29.6895	0.8034	4.1599	0.7061	0.1022	3.6285	4.4315	4.1971	4.7964	4.3627
Average	ACC	0.6898	0.6616	0.6819	0.6554	0.6901	0.6502	0.6802	0.6506	0.6718	**0.7248**	**0.7040**	**0.7266**	**0.7271**
	SD	0.0674	0.0862	0.0821	0.0826	0.0737	0.0900	0.0729	0.0867	0.0604	0.0649	0.0825	0.0794	0.0711
	NUM	85	63	85	116	63	98	70	80	108	85	50	65	88
	Time/s	0.7782	141.9426	10.4410	76.2562	0.7834	2.1599	0.4410	0.0637	2.8683	3.8699	4.3634	4.3786	4.2730

Remarks: ACC, SD, and NUM denote the average classification accuracy, standard deviation, and the number of selected features, respectively. The underlined and bold text indicates that the method achieves the optimal accuracy. PriFS^{POS}, PriFS^{NEG}, PriFS^{DIF}, and PriFS^{AVG} are the proposed methods using different prior connectivity information.

Table S2. Comparison of classification results for feature selection methods based on LDA classifier

		ReliefF	$l_{2,1}$-RLDA	S^{2}DFS	SIFE	ANOVA	Lasso	RFS	FS20	ALLDA	PriFS^{POS}	PriFS^{NEG}	PriFS^{DIF}	PriFS^{AVG}
COBRE	ACC	0.6752	0.6561	0.6624	0.5669	0.6752	0.6242	0.6688	0.6624	0.7070	**0.7389**	**0.7070**	**0.7325**	**0.7325**
	SD	0.0859	0.1261	0.1231	0.0932	0.1233	0.1595	0.1051	0.1102	0.1158	0.0896	0.0984	0.1190	0.0788
	NUM	40	40	30	48	50	60	70	20	60	100	50	80	30
	Time/s	0.4396	83.6036	12.2380	70.2803	0.7575	0.4648	0.1533	0.0224	2.2420	4.2567	5.0330	4.1198	5.8267
MPRC	ACC	0.6495	0.6366	0.6443	0.6108	0.6778	0.5773	0.6675	0.6392	0.6495	0.6727	0.6753	**0.6856**	**0.6933**
	SD	0.0375	0.0776	0.0822	0.0635	0.0714	0.0691	0.0762	0.0589	0.0704	0.0656	0.0860	0.0583	0.0471
	NUM	20	50	30	36	20	20	20	50	50	20	30	20	40
	Time/s	1.2962	160.0539	15.2101	111.4586	0.8027	2.5717	0.5878	0.0861	7.5310	4.3610	3.6900	3.9668	4.3144
FBIRN	ACC	0.7473	0.7260	0.7117	0.6940	0.7473	0.7082	0.7616	0.7046	0.7224	**0.7900**	**0.7616**	**0.7829**	**0.8078**
	SD	0.0570	0.0723	0.0882	0.0781	0.0613	0.0853	0.0875	0.0543	0.0601	0.0753	0.0530	0.0608	0.0812
	NUM	80	40	50	107	20	100	30	20	70	40	90	20	30
	Time/s	0.2723	142.7261	12.5939	76.7577	0.7699	1.3749	0.3170	0.0440	2.1974	4.1261	3.6956	4.5082	4.1145
B-SNIP	ACC	0.6706	0.6396	**0.6993**	0.5871	0.6850	0.6086	0.6563	0.6348	0.6468	0.6921	0.6683	0.6969	0.6826
	SD	0.0854	0.0728	0.0563	0.0661	0.0654	0.0556	0.0454	0.0718	0.0787	0.0644	0.0873	0.0525	0.0624
	NUM	50	50	50	174	40	20	20	90	30	60	20	70	50
	Time/s	1.3406	181.3869	1.7219	112.7093	0.8034	3.9733	0.7061	0.1022	2.5539	4.7219	4.3710	2.1767	4.3627
Average	ACC	0.6857	0.6646	0.6794	0.6147	0.6963	0.6296	0.6886	0.6603	0.6814	**0.7234**	**0.7030**	**0.7245**	**0.7290**
	SD	0.0664	0.0872	0.0874	0.0752	0.0803	0.0924	0.0785	0.0738	0.0812	0.0737	0.0812	0.0726	0.0674
	NUM	48	45	40	91	33	50	35	45	53	55	48	48	38
	Time/s	0.8372	141.9426	10.4410	92.8015	0.7834	2.0962	0.4410	0.0637	3.6311	4.3664	4.1974	3.6929	4.6546

Remarks: ACC, SD, and NUM denote the average classification accuracy, standard deviation, and the number of selected features, respectively. The underlined and bold text indicates that the method achieves the optimal accuracy. PriFS^{POS}, PriFS^{NEG}, PriFS^{DIF}, and PriFS^{AVG} are the proposed methods using different prior connectivity information.

Table S3. Significance analysis of classification accuracy between the proposed PriFS^{NEG} method and each comparison method based on SVM classifier

		ReliefF	$l_{2,1}$-RLDA	S^{2}DFS	SIFE	ANOVA	Lasso	RFS	FS20	ALLDA
**COBRE**	**P-value**	6.50e-05	5.44e-04	1.15e-04	1.05e-10	1.64e-01	2.91e-13	7.74e-02	1.38e-05	3.99e-01
	**T-value**	4.11	3.53	3.96	6.93	1.40	7.98	1.78	4.49	0.85
**MPRC**	**P-value**	3.03e-06	1.17e-31	1.89e-07	6.24e-46	1.16e-04	3.96e-96	5.73e-04	2.52e-10	5.03e-03
	**T-value**	4.74	12.83	5.31	16.32	3.89	28.26	3.47	6.50	2.82
**FBIRN**	**P-value**	1.94e-07	6.06e-09	2.86e-22	3.71e-09	1	1.17e-10	4.84e-01	4.61e-69	5.52e-17
	**T-value**	5.34	6.00	10.59	6.09	0	6.70	-0.70	23.75	8.94
**B-SNIP**	**P-value**	2.87e-01	2.50e-29	1	2.88e-61	1.10e-11	9.47e-54	1.36e-08	6.39e-07	2.67e-10
	**T-value**	1.07	12.16	3.73e-15	19.63	-6.99	17.93	5.79	5.06	6.47

Remarks: Underlined text indicates that the PriFS^{NEG} method significantly outperforms the comparison methods in terms of classification accuracy ($p \leq 0.05$ with Bonferroni correction).

Table S4. Significance analysis of classification accuracy between the proposed PriFS^{DIF} method and each comparison method based on SVM classifier

		ReliefF	$l_{2,1}$-RLDA	S^{2}DFS	SIFE	ANOVA	Lasso	RFS	FS20	ALLDA
COBRE	P-value	4.44e-07	1.83e-09	1.92e-10	5.75e-09	4.06e-03	1.84e-10	4.72e-04	1.89e-08	2.14e-03
	T-value	5.27	6.39	6.82	6.17	2.92	6.82	3.57	5.93	3.12
MPRC	P-value	6.69e-10	6.88e-37	1.40e-16	1.78e-51	6.46e-13	8.85e-123	2.74e-10	6.44e-13	1.91e-09
	T-value	6.33	14.14	8.65	17.63	7.44	35.22	6.48	7.44	6.15
FBIRN	P-value	3.54e-20	2.02e-28	4.63e-30	5.15e-30	4.74e-08	7.92e-31	5.94e-05	4.21e-83	1.33e-24
	T-value	9.95	12.39	12.86	12.85	5.61	13.08	4.08	27.98	11.29
B-SNIP	P-value	7.03e-06	2.68e-48	5.04e-13	1.68e-97	5.06e-03	3.33e-91	1.86e-31	4.82e-119	4.40e-76
	T-value	4.55	16.69	7.46	27.90	-2.82	26.43	12.69	33.15	22.96

Remarks: Underlined text indicates that the PriFS^{DIF} method significantly outperforms the comparison methods in terms of classification accuracy ($p \leq 0.05$ with Bonferroni correction).

Table S5. Significance analysis of classification accuracy between the proposed PriFS^{AVG} method and each comparison method based on SVM classifier

		ReliefF	$l_{2,1}$-RLDA	S^{2}DFS	SIFE	ANOVA	Lasso	RFS	FS20	ALLDA
COBRE	P-value	3.02e-14	9.14e-15	3.04e-13	9.63e-20	4.99e-06	3.13e-21	3.99e-11	1.68e-18	6.48e-05
	T-value	8.37	8.58	7.98	10.46	4.73	11.01	7.11	10.00	4.11
MPRC	P-value	4.30e-11	1.52e-61	6.37e-28	2.18e-65	8.60e-12	3.97e-190	3.27e-07	1.60e-10	7.41e-04
	T-value	6.79	19.98	11.87	20.88	7.04	56.91	5.20	6.57	3.40
FBIRN	P-value	4.24e-24	3.89e-48	4.28e-32	2.64e-26	1.65e-11	7.18e-34	1.12e-09	3.60e-68	3.24e-55
	T-value	11.14	17.86	13.43	11.79	7.02	13.93	6.30	23.49	19.81
B-SNIP	P-value	1.07e-01	1.07e-38	1.23e-01	1.91e-209	2.66e-11	3.28e-108	1.64e-19	1.67e-21	8.08e-34
	T-value	1.62	14.46	1.55	60.71	-6.85	30.47	9.50	10.07	13.28

Remarks: Underlined text indicates that the PriFS^{AVG} method significantly outperforms the comparison methods in terms of classification accuracy ($p \leq 0.05$ with Bonferroni correction).

Table S6. Group differences of the top-10 important FNC features selected by PriFS^{POS}on different datasets, including COBRE, MPRC, FBIRN, and B-SNIP datasets

FNCs (FN ID, Domain)	COBRE			MPRC			FBIRN			B-SNIP		
	P-value (HC vs. SZ)	T-value (HC vs. SZ)	FNC (HC/ SZ)	P-value (HC vs. SZ)	T-value (HC vs. SZ)	FNC (HC/ SZ)	P-value (HC vs. SZ)	T-value (HC vs. SZ)	FNC (HC/ SZ)	P-value (HC vs. SZ)	T-value (HC vs. SZ)	FNC (HC/ SZ)
**Thalamus (45, SC) - Cerebellum (18, CB)**	8.31e-06	4.61	0.19/ 0.02	3.00e-09	6.07	0.19/ 0.06	3.52e-13	7.64	0.19/ 0	4.21e-09	6	0.19/ 0.07
**Thalamus (45, SC) - STG (21, AU)**	1.59e-05	-4.46	0.13/ 0.32	4.39e-09	-6.01	0.13/ 0.28	1.54e-15	-8.46	0.13/ 0.37	1.21e-06	-4.93	0.13/ 0.24
**Thalamus (45, SC) - PoCG (3, SM)**	1.73e-05	-4.44	0.14/ 0.3	1.25e-04	-3.87	0.14/ 0.23	1.18e-12	-7.45	0.14/ 0.34	1.80e-08	-5.74	0.14/ 0.26
**Subthalamus (53, SC) - Cerebellum (7, CB)**	1.24e-04	3.94	0.17/ 0.04	9.56e-06	4.49	0.17/ 0.09	6.98e-13	7.53	0.17/ -0.02	1.22e-06	4.92	0.17/ 0.08
**Subthalamus (53, SC) - STG (21, AU)**	6.68e-04	-3.47	0.22/ 0.34	9.34e-07	-4.99	0.22/ 0.33	9.23e-10	-6.34	0.22/ 0.38	2.07e-04	-3.74	0.22/ 0.29
**Subthalamus (53, SC) - Cerebellum (4, CB)**	5.95e-03	2.79	0.23/ 0.14	7.59e-04	3.39	0.23/ 0.16	2.58e-10	6.56	0.23/ 0.06	1.64e-06	4.86	0.23/ 0.14
**Cuneus (15, VS) - LingualG (8, VS)**	1.39e-03	3.25	0.81/ 0.71	6.17e-03	2.75	0.81/ 0.76	6.69e-05	4.05	0.81/ 0.72	2.65e-06	4.76	0.81/ 0.74
**L PoCG (9, SM) - MTG (62, VS)**	2.33e-04	3.77	0.5/ 0.38	1.29e-03	3.24	0.5/ 0.43	3.00e-05	4.24	0.5/ 0.39	1.05e-03	3.3	0.5/ 0.43
**R MOG (12, VS) - IOG (20, VS)**	2.01e-02	2.35	0.64/ 0.59	1.56e-02	2.43	0.64/ 0.59	8.91e-06	4.53	0.64/ 0.56	9.92e-05	3.93	0.64/ 0.58
**Cuneus (15, VS) - R MOG (12, VS)**	3.11e-02	2.18	0.54/ 0.48	7.86e-02	1.76	0.54/ 0.51	3.18e-06	4.76	0.54/ 0.43	5.36e-04	3.49	0.54/ 0.48

Remarks: For each dataset, two-sample t-tests are used to examine the group differences for each selected FNC feature, and the p-values and T-values between HC group and SZ group are shown. Here, FN denotes the functional network. The brain is divided into seven brain functional domains, including subcortical (SC), auditory (AU), sensorimotor (SM), visual (VS), cognitive-control (CC), default-mode (DM), and cerebellar (CB) domains. STG: Superior temporal gyrus; PoCG: Postcentral gyrus; LingualG: Lingual gyrus; MTG: Middle temporal gyrus; L PoCG: Left postcentral gyrus; R MOG: Right middle occipital gyrus; IOG: Inferior occipital gyrus.

Table S7. Group differences of the top-10 important FNC features selected by PriFS^{NEG}on different datasets, including COBRE, MPRC, FBIRN, and B-SNIP datasets

FNCs (FN ID, Domain)	COBRE			MPRC			FBIRN			B-SNIP		
	P-value (HC vs. SZ)	T-value (HC vs. SZ)	FNC (HC/ SZ)	P-value (HC vs. SZ)	T-value (HC vs. SZ)	FNC (HC/ SZ)	P-value (HC vs. SZ)	T-value (HC vs. SZ)	FNC (HC/ SZ)	P-value (HC vs. SZ)	T-value (HC vs. SZ)	FNC (HC/ SZ)
**Thalamus (45, SC) - Cerebellum (13, CB)**	1.80e-06	4.96	-0.01/ -0.18	8.30e-07	5.01	-0.01/ -0.11	1.02e-12	7.47	-0.01/ -0.21	7.17e-08	5.49	-0.01/ -0.11
**Caudate (69, SC) - STG (21, AU)**	4.68e-05	-4.19	-0.22/ -0.09	6.28e-12	-7.09	-0.22/ -0.08	3.55e-07	-5.22	-0.22/ -0.1	1.61e-06	-4.87	-0.22/ -0.13
**Thalamus (45, SC) - MTG (62, VS)**	2.24e-05	-4.37	-0.3/ -0.14	2.52e-03	-3.04	-0.3/ -0.24	2.52e-15	-8.39	-0.3/ -0.09	3.25e-05	-4.2	-0.3/ -0.22
**Thalamus (45, SC) - CalcarineG (16, VS)**	1.74e-05	-4.43	-0.31/ -0.16	2.08e-04	-3.74	-0.31/ -0.23	2.08e-15	-8.41	-0.31/ -0.09	1.61e-03	-3.17	-0.31/ -0.25
**Subthalamus (53, SC) - Cerebellum (18, CB)**	3.03e-04	3.7	-0.15/ -0.27	4.61e-06	4.65	-0.15/ -0.24	2.36e-09	6.17	-0.15/ -0.31	6.32e-05	4.04	-0.15/ -0.22
**R PoCG (11, SM) - Cerebellum (7, CB)**	2.30e-03	-3.1	-0.31/ -0.21	2.57e-04	-3.69	-0.31/ -0.25	3.32e-08	-5.68	-0.31/ -0.18	3.47e-05	-4.19	-0.31/ -0.23
**Caudate (69, SC) - MTG (62, VS)**	5.24e-04	-3.54	-0.45/ -0.36	4.43e-05	-4.13	-0.45/ -0.39	6.49e-09	-5.99	-0.45/ -0.33	3.80e-03	-2.91	-0.45/ -0.4
**Subthalamus (53, SC) - R MOG (12, VS)**	4.14e-05	-4.22	-0.21/ -0.08	2.90e-03	-3	-0.21/ -0.16	1.35e-11	-7.06	-0.21/ -0.06	2.94e-02	-2.19	-0.21/ -0.18
**Subthalamus (53, SC) - L PoCG (9, SM)**	8.02e-05	-4.05	-0.26/ -0.13	3.41e-04	-3.61	-0.26/ -0.19	1.41e-08	-5.85	-0.26/ -0.12	4.62e-03	-2.85	-0.26/ -0.21
**Thalamus (45, SC) - R MOG (12, VS)**	4.56e-06	-4.75	-0.19/ -0.05	1.03e-01	-1.63	-0.19/ -0.16	4.76e-11	-6.85	-0.19/ -0.03	1.26e-02	-2.51	-0.19/ -0.15

Remarks: For each dataset, two-sample t-tests are used to examine the group differences for each selected FNC feature, and the p-values and T-values between HC group and SZ group are shown. Here, FN denotes the functional network. The brain is divided into seven brain functional domains, including subcortical (SC), auditory (AU), sensorimotor (SM), visual (VS), cognitive-control (CC), default-mode (DM), and cerebellar (CB) domains. STG: Superior temporal gyrus; MTG: Middle temporal gyrus; CalcarineG: Calcarine gyrus; R MOG: Right middle occipital gyrus; L PoCG: Left postcentral gyrus; R PoCG: Right postcentral gyrus.

Table S8. Group differences of the top-10 important FNC features selected by PriFS^{DIF} on different datasets, including COBRE, MPRC, FBIRN, and B-SNIP datasets

FNCs (FN ID, Domain)	COBRE			MPRC			FBIRN			B-SNIP		
	P-value (HC vs. SZ)	T-value (HC vs. SZ)	FNC (HC/ SZ)	P-value (HC vs. SZ)	T-value (HC vs. SZ)	FNC (HC/ SZ)	P-value (HC vs. SZ)	T-value (HC vs. SZ)	FNC (HC/ SZ)	P-value (HC vs. SZ)	T-value (HC vs. SZ)	FNC (HC/ SZ)
**Thalamus (45, SC) - Cerebellum (18, CB)**	8.31e-06	4.61	0.19/ 0.02	3.00e-09	6.07	0.19/ 0.06	3.52e-13	7.64	0.19/ 0	4.21e-09	6	0.19/ 0.07
**Thalamus (45, SC) - STG (21, AU)**	1.59e-05	-4.46	0.13/ 0.32	4.39e-09	-6.01	0.13/ 0.28	1.54e-15	-8.46	0.13/ 0.37	1.21e-06	-4.93	0.13/ 0.24
**Thalamus (45, SC) - PoCG (3, SM)**	1.73e-05	-4.44	0.14/ 0.3	1.25e-04	-3.87	0.14/ 0.23	1.18e-12	-7.45	0.14/ 0.34	1.80e-08	-5.74	0.14/ 0.26
**Caudate (69, SC) - STG (21, AU)**	4.68e-05	-4.19	-0.22/ -0.09	6.28e-12	-7.09	-0.22/ -0.08	3.55e-07	-5.22	-0.22/ -0.1	1.61e-06	-4.87	-0.22/ -0.13
**Subthalamus (53, SC) - Cerebellum (7, CB)**	1.24e-04	3.94	0.17/ 0.04	9.56e-06	4.49	0.17/ 0.09	6.98e-13	7.53	0.17/ -0.02	1.22e-06	4.92	0.17/ 0.08
**Caudate (69, SC) - Cerebellum (18, CB)**	4.33e-04	3.6	0.3/ 0.2	5.60e-10	6.36	0.3/ 0.18	1.84e-07	5.35	0.3/ 0.17	1.38e-07	5.36	0.3/ 0.2
**Thalamus (45, SC) - MTG (62, VS)**	2.24e-05	-4.37	-0.3/ -0.14	2.52e-03	-3.04	-0.3/ -0.24	2.52e-15	-8.39	-0.3/ -0.09	3.25e-05	-4.2	-0.3/ -0.22
**Thalamus (45, SC) - CalcarineG (16, VS)**	1.74e-05	-4.43	-0.31/ -0.16	2.08e-04	-3.74	-0.31/ -0.23	2.08e-15	-8.41	-0.31/ -0.09	1.61e-03	-3.17	-0.31/ -0.25
**Thalamus (45, SC) - SMA (84, CC)**	1.83e-03	-3.17	0.06/ 0.16	5.26e-04	-3.5	0.06/ 0.12	3.22e-15	-8.35	0.06/ 0.23	1.94e-03	-3.12	0.06/ 0.11
**Subthalamus (53, SC) - R MOG (12, VS)**	4.14e-05	-4.22	-0.21/ -0.08	2.90e-03	-3	-0.21/ -0.16	1.35e-11	-7.06	-0.21/ -0.06	2.94e-02	-2.19	-0.21/ -0.18

Remarks: For each dataset, two-sample t-tests are used to examine the group differences for each selected FNC feature, and the p-values and T-values between HC group and SZ group are shown. Here, FN denotes the functional network. The brain is divided into seven brain functional domains, including subcortical (SC), auditory (AU), sensorimotor (SM), visual (VS), cognitive-control (CC), default-mode (DM), and cerebellar (CB) domains. STG: Superior temporal gyrus; PoCG: Postcentral gyrus; MTG: Middle temporal gyrus; CalcarineG: Calcarine gyrus; SMA: Supplementary motor area; R MOG: Right middle occipital gyrus.

Table S9. Group differences of the top-10 important FNC features selected by PriFS^{AVG} on different datasets, including COBRE, MPRC, FBIRN, and B-SNIP datasets

FNCs (FN ID, Domain)	COBRE			MPRC			FBIRN			B-SNIP		
	P-value (HC vs. SZ)	T-value (HC vs. SZ)	FNC (HC/ SZ)	P-value (HC vs. SZ)	T-value (HC vs. SZ)	FNC (HC/ SZ)	P-value (HC vs. SZ)	T-value (HC vs. SZ)	FNC (HC/ SZ)	P-value (HC vs. SZ)	T-value (HC vs. SZ)	FNC (HC/ SZ)
**Thalamus (45, SC) - STG (21, AU)**	1.59e-05	-4.46	0.13/ 0.32	4.39e-09	-6.01	0.13/ 0.28	1.54e-15	-8.46	0.13/ 0.37	1.21e-06	-4.93	0.13/ 0.24
**Thalamus (45, SC) - Cerebellum (13, CB)**	1.80e-06	4.96	-0.01/ -0.18	8.30e-07	5.01	-0.01/ -0.11	1.02e-12	7.47	-0.01/ -0.21	7.17e-08	5.49	-0.01/ -0.11
**Thalamus (45, SC) - PoCG (3, SM)**	1.73e-05	-4.44	0.14/ 0.3	1.25e-04	-3.87	0.14/ 0.23	1.18e-12	-7.45	0.14/ 0.34	1.80e-08	-5.74	0.14/ 0.26
**Thalamus (45, SC) - MTG (62, VS)**	2.24e-05	-4.37	-0.3/ -0.14	2.52e-03	-3.04	-0.3/ -0.24	2.52e-15	-8.39	-0.3/ -0.09	3.25e-05	-4.2	-0.3/ -0.22
**Thalamus (45, SC) - CalcarineG (16, VS)**	1.74e-05	-4.43	-0.31/ -0.16	2.08e-04	-3.74	-0.31/ -0.23	2.08e-15	-8.41	-0.31/ -0.09	1.61e-03	-3.17	-0.31/ -0.25
**Cuneus (15, VS) - LingualG (8, VS)**	1.39e-03	3.25	0.81/ 0.71	6.17e-03	2.75	0.81/ 0.76	6.69e-05	4.05	0.81/ 0.72	2.65e-06	4.76	0.81/ 0.74
**L PoCG (9, SM) - MTG (62, VS)**	2.33e-04	3.77	0.5/ 0.38	1.29e-03	3.24	0.5/ 0.43	3.00e-05	4.24	0.5/ 0.39	1.05e-03	3.3	0.5/ 0.43
**Caudate (69, SC) - CalcarineG (16, VS)**	1.47e-01	-1.46	-0.77/ -0.73	3.29e-04	-3.62	-0.77/ -0.71	5.82e-10	-6.42	-0.77/ -0.62	1.56e-02	-2.43	-0.77/ -0.73
**R MOG (12, VS) - IOG (20, VS)**	2.01e-02	2.35	0.64/ 0.59	1.56e-02	2.43	0.64/ 0.59	8.91e-06	4.53	0.64/ 0.56	9.92e-05	3.93	0.64/ 0.58
**Cuneus (15, VS) - R MOG (12, VS)**	3.11e-02	2.18	0.54/ 0.48	7.86e-02	1.76	0.54/ 0.51	3.18e-06	4.76	0.54/ 0.43	5.36e-04	3.49	0.54/ 0.48

Remarks: For each dataset, two-sample t-tests are used to examine the group differences for each selected FNC feature, and the p-values and T-values between HC group and SZ group are shown. Here, FN denotes the functional network. The brain is divided into seven brain functional domains, including subcortical (SC), auditory (AU), sensorimotor (SM), visual (VS), cognitive-control (CC), default-mode (DM), and cerebellar (CB) domains. STG: Superior temporal gyrus; PoCG: Postcentral gyrus; MTG: Middle temporal gyrus; CalcarineG: Calcarine gyrus; LingualG: Lingual gyrus; R MOG: Right middle occipital gyrus; IOG: Inferior occipital gyrus. L PoCG: Left postcentral gyrus.

Table S10. Top-10 functional network connectivity identified by our proposed PriFS^{POS}, PriFS^{NEG}, PriFS^{DIF}, and PriFS^{AVG} methods across the four datasets

FNCs (FN ID, Domain)	PriFS^{POS}	PriFS^{NEG}	PriFS^{DIF}	PriFS^{AVG}	Amplitude
**Thalamus (45, SC) - Cerebellum (18, CB)**	●		●		Decrease
**Subthalamus (53, SC) - Cerebellum (7, CB)**	●		●		Decrease
**Cuneus (15, VS) - LingualG (8, VS)**	●			●	Decrease
**L PoCG (9, SM) - MTG (62, VS)**	●			●	Decrease
**R MOG (12, VS) - IOG (20, VS)**	●			●	Decrease
**Cuneus (15, VS) - R MOG (12, VS)**	●			●	Decrease
**Subthalamus (53, SC) - Cerebellum (4, CB)**	●				Decrease
**Subthalamus (53, SC) - STG (21, AU)**	●				Increase
**Thalamus (45, SC) - STG (21, AU)**	●		●	●	Increase
**Thalamus (45, SC) - PoCG (3, SM)**	●		●	●	Increase
**Thalamus (45, SC) - MTG (62, VS)**		●	●	●	Decrease
**Thalamus (45, SC) - CalcarineG (16, VS)**		●	●	●	Decrease
**Caudate (69, SC) - STG (21, AU)**		●	●		Decrease
**Subthalamus (53, SC) - R MOG (12, VS)**		●	●		Decrease
**R PoCG (11, SM) - Cerebellum (7, CB)**		●			Decrease
**Caudate (69, SC) - MTG (62, VS)**		●			Decrease
**Subthalamus (53, SC) - L PoCG (9, SM)**		●			Decrease
**Thalamus (45, SC) - R MOG (12, VS)**		●			Decrease
**Thalamus (45, SC) - Cerebellum (13, CB)**		●		●	Increase
**Subthalamus (53, SC) - Cerebellum (18, CB)**		●			Increase
**Caudate (69, SC) - Cerebellum (18, CB)**			●		Decrease
**Thalamus (45, SC) - SMA (84, CC)**			●		Increase
**Caudate (69, SC) - CalcarineG (16, VS)**				●	Decrease

Remarks: The brain is divided into seven brain functional domains, including subcortical (SC), auditory (AU), sensorimotor (SM), visual (VS), cognitive-control (CC), default-mode (DM), and cerebellar (CB) domains. FN denotes the functional network. CalcarineG: Calcarine gyrus; IOG: Inferior occipital gyrus; LingualG: Lingual gyrus; MTG: Middle temporal gyrus; R MOG: Right middle occipital gyrus; PoCG: Postcentral gyrus; L PoCG: Left postcentral gyrus; R PoCG: Right postcentral gyrus; SMA: Supplementary motor area; STG: Superior temporal gyrus. Amplitude represents the change of the selected FNC in SZ group relative to HC group.
